# Stress-induced preference for antioxidants by *Drosophila*

**DOI:** 10.1073/pnas.2512852122

**Published:** 2025-09-18

**Authors:** Gayoung Hwang, Dae-Wook Yang, Terezia Klaudia Geisseova, Hae A. Kim, Yangkyun Oh, Greg S. B. Suh

**Affiliations:** ^a^Department of Biological Sciences, Korea Advanced Institute of Science and Technology, Daejeon 34141, Republic of Korea; ^b^Department of Biological Sciences, Ulsan National Institute of Science and Technology, Ulsan 44919, Republic of Korea; ^c^Korea Advanced Institute of Science and Technology Analysis Center for Research Advancement, Korea Advanced Institute of Science and Technology, Daejeon 34141, Republic of Korea; ^d^Department of Life Science, College of Natural Sciences, Ewha Womans University, Seoul 03760, Republic of Korea

**Keywords:** stress-induced attraction to antioxidant, self-medication, vitamin C, reactive oxygen species (ROS), *Drosophila*

## Abstract

In an ever-changing environment, animals make optimal decisions to ensure their well-being. Faced with limited food sources, they often seek foods that provide the nutrients they require to maintain homeostasis. Under extreme circumstances (e.g., infection), they may exhibit ingestive behaviors, such as seeking out substances (e.g., toxins) that suggest self-medication. Few studies, however, have investigated the mechanisms that ensue self-medication. Here, we report the selective intake of antioxidants by *Drosophila melanogaster* during a period of heat stress or sleep deprivation. This preference was alleviated by prefeeding them vitamin C or dehydroascorbic acid (DHA) before exposure to stress. Heat stress led to an increase in reactive oxygen species (ROS) levels in the gut, which was alleviated by the intake of vitamin C. Heat stress reduced vitamin C in hemolymph, whereas the consumption of vitamin C or DHA increased it. Furthermore, the intake of vitamin C ameliorated the intestinal barrier dysfunction and extended the survival of flies that had been exposed to chronic heat stress. The heat-induced preference for vitamin C appears to develop independently of the known peripheral chemosensory receptors for this micronutrient. We propose that fruit flies possess an interoceptive mechanism that mediates the detection of vitamin C to overcome environmental challenges.

## Significance

Animals possess an innate ability to detect chemical cues associated with beneficial substances in the environment. Chimpanzees, for example, are able to identify a particular herb that can fight off parasites during infection—a phenomenon called “self-medication.” Other examples of self-medication have been observed, but the mechanisms by which animals respond to needed chemicals are unclear. Here, we report that fruit flies selectively consume antioxidants following heat stress or sleep deprivation. Remarkably, antioxidant intake significantly ameliorated stress-induced increases in ROS levels, gut leakage, and reduced survival. Furthermore, the preference for vitamin C appears to be independent of the known peripheral chemosensors for this micronutrient, suggesting the involvement of an interoceptive sensing mechanism.

Animals encounter countless challenges in the wild. Among them is the need to develop a strategy to survive and produce offspring. Because of limited food sources in the wild, animals must use their peripheral taste and olfactory systems to locate foods rich in the nutrients they require to do so (1, 2). Recent studies have uncovered the identities and characteristics of several interoceptive nutrient-sensing systems that mediate the detection of essential nutrients, particularly those of which the animal had been deprived (3–8). Many interoceptive sensing systems that respond to macronutrients (carbohydrates, proteins, and fats) independently of taste or olfactory inputs have been defined (3–6, 9–11), but systems capable of responding to micronutrients have been less explored (7, 8).

Furthermore, pathogenic infection or oxidative stress can drive animals to seek specific chemicals (e.g., toxins) through a behavior known as “self-medication” (12–17). This unique foraging behavior has been observed in many animal species: Chimpanzees, for instance, attempt to relieve symptoms of parasite infection by sucking the pith of the herb known as *Vernonia amygdalina*, despite its intense bitterness (12, 13); Gouldian finches, which usually eat white sorghum at moderate temperatures, switch to red sorghum when the temperature drops, possibly because the greater amount of polyphenols in red sorghum helps minimize the oxidative stress associated with the large shift in temperature (14); infected tiger moth caterpillars have an altered taste for pyrrolizidine alkaloid, a plant toxin, that injures their predator and parasitoids (15). Unfortunately, little attempt has been made to uncover the mechanistic details of self-medication.

Micronutrients are required in small quantities but are essential for maintaining health and proper metabolism (18, 19). Micronutrients include vitamins, minerals, and metals, many of which must be provided through exogenous supplements or food sources because they cannot be synthesized in the body (20). These include vitamin C—a water-soluble vitamin whose functions include scavenging free radicals, biosynthesizing collagen, and enhancing iron absorption (21). The biosynthesis of vitamin C requires the presence of *GULO,* a gene responsible for the production of L-gluconolactone oxidase, the enzyme that catalyzes the last step in the metabolic pathway for the biosynthesis of vitamin C (22, 23). However, humans cannot synthesize vitamin C because the *GULO* gene is mutated in humans and is no longer functional (24). For this reason, we must rely on fruits and vegetables as sources of vitamin C. In fruit flies, the ortholog of the *GULO* gene, which is responsible for synthesizing vitamin C, is absent. Recent studies, however, suggested that vitamin C might be produced either by an enzyme similar to *GULO* or through an alternative biosynthetic pathway (25, 26).

Reactive oxygen species (ROS) play important roles in physiological processes; however, excessive levels can lead to oxidative stress that can damage macromolecules such as DNA, proteins, and lipids (27, 28). Living organisms use antioxidants to defend their organs and themselves against damage caused by oxidative stress (29, 30). Vitamin C, as an antioxidant, has a low reduction potential (*E_0_* = 282 mV; pH 7), indicating a strong tendency to donate electrons. By contrast, molecules with higher reduction potentials, such as superoxide (*E_0_* = 940 mV; pH 7), are more likely to accept electrons, allowing vitamin C to efficiently neutralize ROS and free radicals by serving as a readily available electron donor (31). Beyond its direct role in scavenging ROS, vitamin C also acts indirectly as an antioxidant by donating electrons to regenerate vitamin E, thereby restoring its antioxidant capacity (32). Despite the important role of antioxidants in maintaining homeostasis in animals, little is known about whether and how stressed animals exhibit behavioral responses to antioxidants.

We found that fruit flies developed an increased preference for vitamin C after being exposed to heat stress or sleep deprivation. This preference was attenuated by prefeeding vitamin C or dehydroascorbic acid (DHA, an oxidized form of vitamin C that is readily converted to active vitamin C in the body) (33–35) before inducing stress. We found that ROS levels were increased in the gut of heat-stressed or sleep-deprived flies, but this increase was attenuated by prefeeding with vitamin C or DHA. Furthermore, the intake of vitamin C or DHA restored intestinal barrier integrity and extended the survival of flies exposed to chronic stress. To investigate whether external sensory cues mediate vitamin C-seeking behavior under stress, we examined taste and olfactory receptor mutants, as well as taste sensitivity using the Proboscis Extension Response (PER) assay (36). These tests revealed no significant differences from controls in their taste sensitivities to vitamin C. By contrast, the Manual Feeding (MAFE) assay (37), which measures postingestive responses, revealed that heat-stressed flies consumed more vitamin C-containing food than control food, whereas control flies consumed less. This suggests that the regulation is likely mediated by internal rather than external sensory mechanisms. These lines of evidence support the existence of a taste-independent mechanism that promotes antioxidant consumption when exposed to stress.

## Results

### *Drosophila* Develops a Preference for vitamin C When Exposed to Stress.

**Fig. 1.**
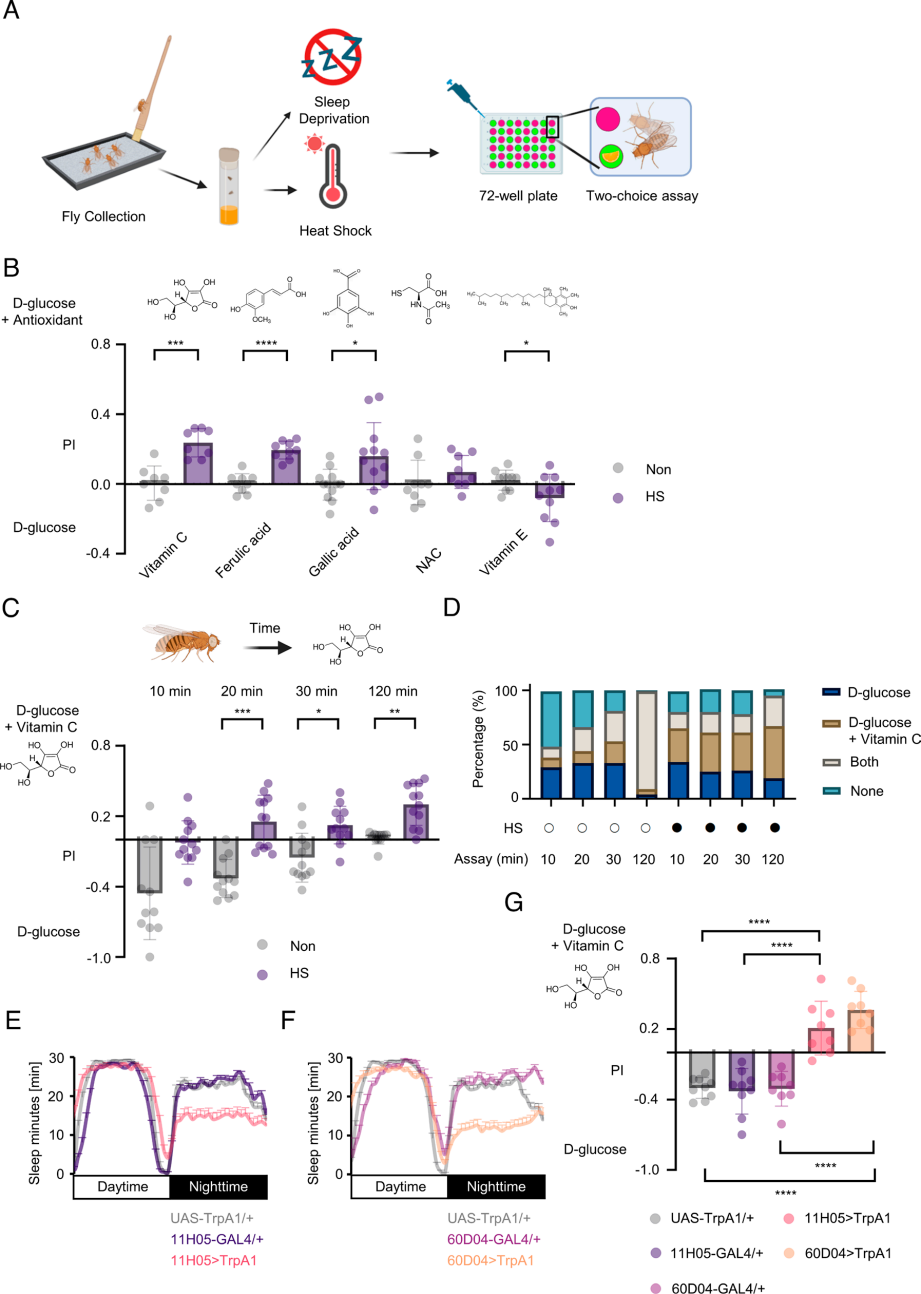
*Drosophila* develops a preference for antioxidants when exposed to stress. (*A*) Schematic illustration of the two-choice assay. (*B* and *C*) Behavioral preferences for antioxidants in heat-stressed flies. (*B*) Flies were given a choice between d-glucose and d-glucose with denoted antioxidant (2 mM) after exposure to heat stress (n = 8 to 12). Non: no heat shock, HS: heat shock (used in this and subsequent figures). (*C*) Development of flies’ behavioral preferences in a two-choice assay conducted at 10, 20, 30, or 120 min after exposure to 1 h heat shock (n = 11 to 12). Filled circles: treated; open circles: untreated (used in this and Fig. 2). (*D*) The percentage of flies that consumed glucose-containing food only, vitamin C-containing food only, both foods, or none in (*C*) (n = 290 to 342). (*E* and *F*) Sleep profile of male flies with activated (*E*) GMR11H05+ neurons (n = 46 to 76) or (*F*) GMR60D04+ neurons (n = 34 to 58), assayed on the 7th day of DAM assay. The white bar represents the light-on phase, and the black bar represents the light-off phase. Quantifications of (*E* and *F*) are shown in *SI Appendix*, Fig. S3 *A* and *B*. (*G*) Two-choice preferences of sleep-deprived male flies between d-glucose and d-glucose with 2 mM vitamin C, assayed on the 7th day (n = 8 to 9). Data are presented as mean ± SD for panels *B*–*D*, and *G*, and mean ± SEM for panels *E* and *F*. Statistical analyses were performed as follows: unpaired two-tailed *t* test for panel *B*; Welch’s ANOVA with Dunnett’s T3’s post hoc test for panel *C*; and one-way ANOVA with Tukey’s post hoc test for panel *G*. *P < 0.05; **P < 0.01; ***P < 0.001; ****P < 0.0001.

To explore the possibility that environmental challenges alter the feeding behavior of flies, we used a two-choice preference assay (38) that allowed heat-stressed or sleep-deprived flies to choose between an antioxidant-containing food and an antioxidant-free food (Fig. 1*A*). The two types of food were labeled using two different colors, and their preferences among several antioxidants were scored by inspecting their abdomens and labella (Fig. 1*A*). The heat-stressed flies demonstrated a preference for some antioxidants—specifically vitamin C (l-ascorbic acid), ferulic acid, and gallic acid—but not others (e.g., N-Acetyl-l-cysteine or vitamin E) (Fig. 1*B*). We focused on vitamin C in subsequent experiments.

When heat-stressed flies were given vitamin C in various concentrations of 1 mM to 20 mM, they showed a preference for a range of concentrations yet exhibited a strong preference for 2 mM vitamin C (*SI Appendix,* Fig. S1*A*). To quantify food intake, we employed both BARCODE-based and absorbance-based methods. The BARCODE-based assay uniquely assigns oligomer-containing food, enabling measurement of consumption via qPCR (39, 40). Using this method, we determined the amount of vitamin C-containing food consumed by heat-stressed flies and confirmed that they exhibited the highest preference for 2 mM vitamin C (*SI Appendix*, Fig. S2*A*). Notably, the intake decreased as the concentration of vitamin C increased, dropping to 0.43 at 8 mM and 0.09 at 20 mM relative to the intake at 2 mM (set as 1) (*SI Appendix*, Fig. S2*B*). Similarly, absorbance-based quantification, using dyes with minimal spectral overlap, allowed measurement of food consumption from flies (41–43). This method corroborated the BARCODE results: Heat-stressed flies exhibited an increased preference for vitamin C, but intake dropped to 0.42 at 20 mM compared to the 2 mM level (*SI Appendix*, Fig. S2 *C* and *D*). These findings raise an intriguing possibility that stressed flies may regulate their vitamin C intake so that the total amount consumed stays within a defined range, converging toward a certain absolute intake level, rather than responding passively to its concentration.

We also subjected flies to heat stress for a duration of 15 min to 60 min. Female flies exhibited an increased preference for vitamin C after 30 min of heat stress, whereas male flies exhibited an increased preference only at 15 min of heat stress (*SI Appendix,* Fig. S1 *B* and *C*). Consistent with these findings, we found that virgin flies and wild-type *Canton-S* flies also preferred vitamin C after experiencing heat stress (*SI Appendix,* Fig. S1 *D–F*). In all, we found that flies preferred vitamin C and other antioxidants under heat stress, regardless of sex, mating status, or strain.

To further investigate the development of vitamin C preference, we conducted our behavioral assay over a shorter time course. During the first 10 min, most control flies did not eat, and those that did tended to avoid the vitamin C-containing food (Fig. 1 *C* and *D*). By contrast, heat-stressed flies showed no initial preference between the two food types (Fig. 1*C*). At 20 min, heat-stressed flies began to exhibit a positive preference for vitamin C-containing food, which became more pronounced over time (Fig. 1*C*). Of note, previous studies have reported that starved flies’ preference to nutritive sugar over non-nutritive sweetener can emerge within a short period, despite this behavior being mediated by a postingestive mechanism (4, 37). At the 120 min mark, control flies consumed both food types equally, resulting in a preference score near zero (Fig. 1 *C* and *D*), whereas heat-stressed flies showed the highest preference for vitamin C (Fig. 1*C*), consistent with earlier findings.

Next, we investigated whether flies exhibit a preference for vitamin C after being exposed to another type of stress. Based on previous studies, the artificial activation of a population of cells labeled by the *11H05-GAL4* or *60D04-GAL4* driver line resulted in a significant loss of sleep in flies (44, 45). We confirmed that flies in which these cells had been artificially activated indeed experienced sleep loss (Fig. 1 *E* and *F* and *SI Appendix*, Fig. S3 *A* and *B*), in accordance with the previous findings (44, 45). Notably, sleep-deprived flies displayed a significant preference for vitamin C in the two-choice assay (Fig. 1*G*). These results suggest that various forms of stress can induce a preference for vitamin C in flies.

### Fruit Flies Are Not Attracted to Either Neutral Vitamin C or Other Acids After Exposure to Heat Stress.

Vitamin C and other antioxidants that were preferred by stressed flies are acidic; 2 mM vitamin C (approximately pH 3.2 to 3.5), 2 mM ferulic acid (pH ~3.62), and 2 mM gallic acid (pH ~3.43). To determine whether flies are attracted to the acidity moiety of antioxidants, we subjected them to choose between d-glucose and acidic d-glucose (pH 3.2 to 3.5). The heat-stressed flies did not show a preference for acidic d-glucose (*SI Appendix,* Fig. S4*A*). To substantiate the hypothesis that heat-stressed flies do not necessarily develop a preference for acidity, we tested whether flies show a preference for other acids (acetic acid, citric acid, and glycolic acid) at 2 mM concentration; The pH of these acids was similar to the pH of 2 mM vitamin C; 2 mM acetic acid (pH ~3.11), 2 mM citric acid (pH ~2.98), and 2 mM glycolic acid (pH ~3.23) (*SI Appendix,* Fig. S4*A*). However, flies did not demonstrate a preference for these acids upon receiving a heat shock (*SI Appendix,* Fig. S4*A*). These results suggest that the heat-shock-induced preference for vitamin C is not manifested by acidity alone.

Next, we have considered the possibility that the preference for antioxidants can be developed by recognizing the difference in pH of the two diets provided in the two-choice assay. After we adjusted the pH of vitamin C, ferulic acid, and gallic acid (preferred antioxidants) to neutral pH (about pH 6 to 7), we tested them in the two-choice assay and found that heat-stressed flies were no longer attracted to neutralized antioxidants (*SI Appendix,* Fig. S4*B*). For nonpreferred antioxidants such as NAC (pH ~3.16) and vitamin E (pH ~5.55), we neutralized NAC to pH 6 to 7 and acidified vitamin E to pH ~3.37, but found that heat-stressed flies did not show a preference for any of these antioxidants that had been altered in their pH (*SI Appendix,* Fig. S4*B*).

Conversely, we subjected flies to select neutralized vitamin C mixed with glucose (pH 6 to 7) versus acidified glucose (pH 3.2 to 3.5) to test whether the pH difference between the two diets in the two-choice arena is important for developing a preference for vitamin C in heat-stress flies. However, these flies did not select neutralized vitamin C, even if the two diets had a difference in pH (*SI Appendix,* Fig. S4*C*). This indicated that flies did not show a preference for vitamin C just because of the pH difference in foods.

It was reported that the stability of vitamin C is dependent on the pH of the solvent (46–48). We measured the levels of vitamin C using liquid chromatography-mass spectrometry (LC–MS) and found that the levels of vitamin C were significantly decreased when it was neutralized (*SI Appendix,* Fig. S4*D*). This is consistent with previous studies (46–48) indicating that the vitamin C levels dropped when its pH was altered. Inferred from these findings, the native form of vitamin C disappears when neutralized (*SI Appendix,* Fig. S4*D*), as its stability might be compromised.

### The Heat-Induced Preference for Vitamin C Is Blunted By Prefeeding Vitamin C or DHA.

**Fig. 2.**
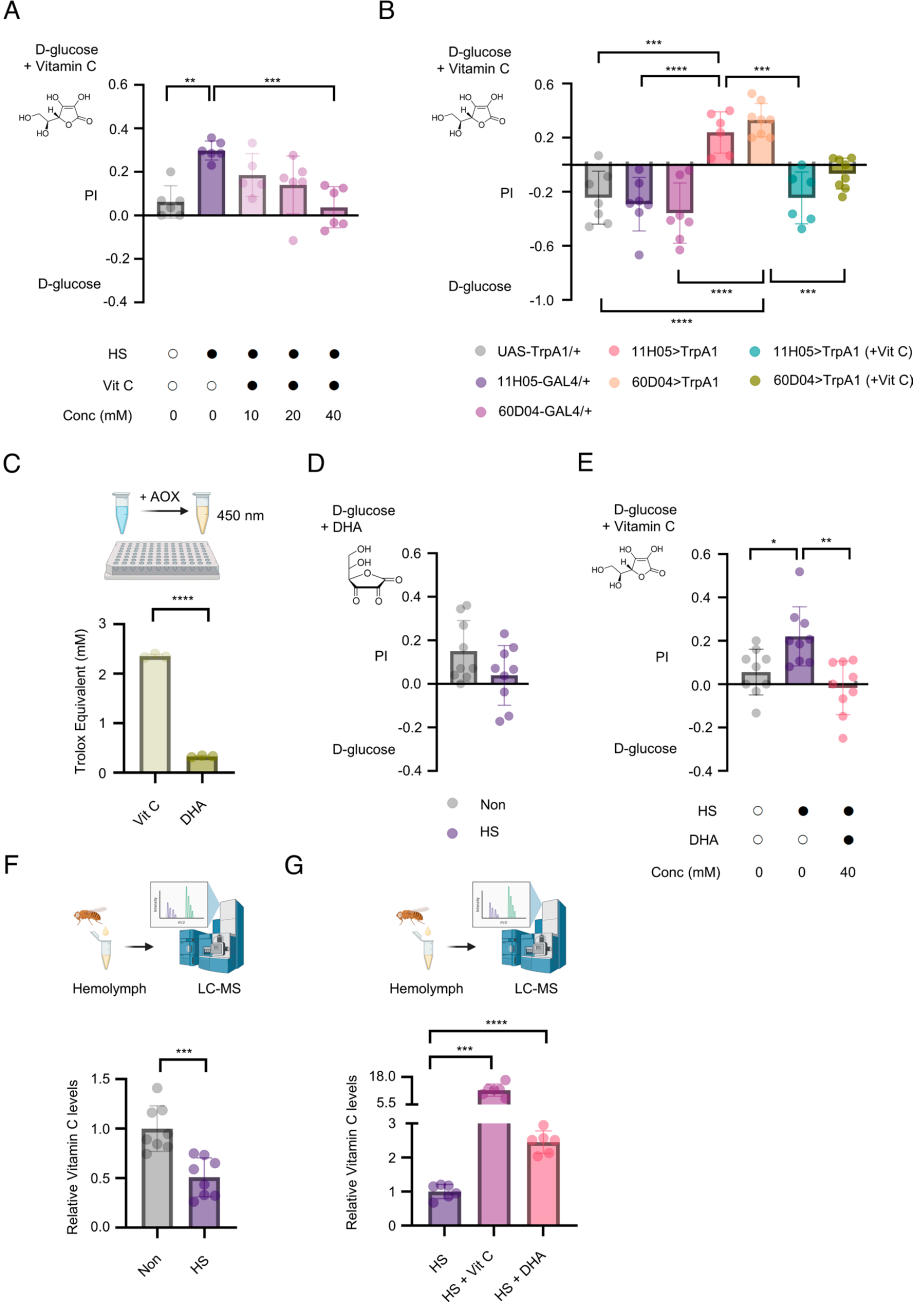
Heat-induced preference for vitamin C is blunted by prefeeding vitamin C or DHA. (*A*) Two-choice preferences (d-glucose versus d-glucose with 2 mM vitamin C) of heat-stressed flies that had been prefed with vitamin C (0, 10, 20, and 40 mM) for 4 d (n = 5 to 6). (*B*) Two-choice preferences for sleep-deprived male flies that had been prefed 10 mM vitamin C, or none for 2 d, assayed on the 7th day (n = 6 to 8). (*C*) Measurement of antioxidant capacity of 2 mM vitamin C and 2 mM DHA using Trolox equivalent antioxidant capacity (TEAC) assay (n = 3). (*D* and *E*) Two-choice preferences of heat-stressed flies (n = 9) (*D*), and heat-stressed flies that had been prefed 40 mM DHA, or none for 4 d (n = 9) (*E*). (*F* and *G*) LC–MS measurement of vitamin C levels in hemolymph of heat-stressed and control flies (n = 8) (*F*), and heat-stressed flies that had been prefed 40 mM vitamin C, 40 mM DHA, or none for 4 d (n = 6) (*G*). Data are presented as mean ± SD. Statistical analyses were performed as follows: one-way ANOVA with Tukey’s post hoc test for panels *A*, *B*, and *E*; Welch’s ANOVA with Dunnett’s T3’s post hoc test for panel *G*; and unpaired two-tailed *t* test for panels *C*, *D*, and *F*. *P < 0.05; **P < 0.01; ***P < 0.001; ****P < 0.0001.

To determine whether feeding flies vitamin C before inducing heat stress affects their feeding preferences, we prefed flies a standard cornmeal food containing different concentrations of vitamin C for 4 d. We found that prefeeding heat-stressed flies with a 40 mM concentration of vitamin C eliminated their preference for vitamin C (Fig. 2*A*). Likewise, prefeeding sleep-deprived flies with vitamin C reduced the preference for vitamin C (Fig. 2*B*).

It is known that vitamin C is converted to DHA (33) when oxidized, and DHA has a weaker antioxidant capacity (49). Consistent with this, we found that the antioxidant capacity of DHA was approximately 14% of the antioxidant capacity of vitamin C (Fig. 2*C*), and heat-stressed flies did not prefer DHA-containing food over DHA-free food (Fig. 2*D*).

DHA can be converted to vitamin C in the body in the presence of glutathione (33–35). In fact, Glutathione S transferase O2 (GstO2) encoded by the *GstO2* gene (*DmGstO1*: *CG6673*) in *Drosophila* has high DHA reductase activity and regulates the vitamin C/DHA ratio (50, 51). Interestingly, prefeeding heat-stressed flies with 40 mM DHA attenuated their preference for vitamin C (Fig. 2*E*). The degree of attenuation was comparable to that seen in flies that had been prefed vitamin C (Fig. 2*A*). We hypothesized that DHA was converted to vitamin C in vivo, which alleviated the need for vitamin C after exposure to heat stress. Consistent with this hypothesis, vitamin C levels in hemolymph were significantly altered by heat stress, as indicated by LC–MS (Fig. 2*F* and also see Fig. 2*G*).

Next, we sought to investigate whether other chemical components of hemolymph were altered following heat shock. To this end, we measured the levels of sugar, protein, and lipid in hemolymph. When we measured the levels of sugar in hemolymph using the anthrone reaction (52), we found that heat shock significantly increased the levels of sugar in hemolymph (*SI Appendix,* Fig. S5*A*). It was suggested that the levels of trehalose, the main source of fuel and energy for insects, increased to effectively resist heat stress (53–55). We measured the concentration of lipids in hemolymph using the sulfo-phospho-vanillin (SPV) reaction (52, 56) and found a reduction in the levels of lipids in hemolymph when flies received heat shock (*SI Appendix,* Fig. S5*B*). It was reported that hemolymphatic lipids were expelled into excretion when flies were challenged with stress (clean injury or infection) to prevent ROS production by hemolymphatic lipid peroxidation (57). Moreover, we measured the levels of protein in hemolymph using the Bradford assay (52) and found that protein levels in hemolymph were also decreased by heat shock (*SI Appendix,* Fig. S5*C*). In addition to measuring the levels of macronutrients in hemolymph, we further determined the levels of antioxidant capacity in hemolymph. We found that when flies received heat shock, antioxidant capacity was increased in the hemolymph (*P* = 0.0716) (*SI Appendix,* Fig. S5*D*). We propose that elevated ROS levels following heat stress trigger an antioxidant response, either by upregulating antioxidant target gene expression or by enhancing antioxidant activity to scavenge ROS (58, 59).

In addition, the intake of DHA, similar to that of vitamin C, increased the levels of vitamin C in hemolymph (Fig. 2*G*). We observed that heat-stressed flies that had been prefed 40 mM vitamin C represented a 12.05-fold increase in the levels of vitamin C in hemolymph compared to heat-stressed flies that had not been prefed (Fig. 2*G*). On the other hand, heat-stress flies that had been prefed with 40 mM DHA held a 2.45-fold increase in the levels of vitamin C in hemolymph compared to those in heat-stressed flies without prefeeding (Fig. 2*G*), confirming our hypothesis and the previous findings (33–35) that DHA is converted to vitamin C in the body. These findings suggest that the physiological levels of vitamin C influence the heat-induced preference for vitamin C in flies.

### Vitamin C Intake Reduces Stress-Induced Increases in ROS Levels of the Gut.

**Fig. 3.**
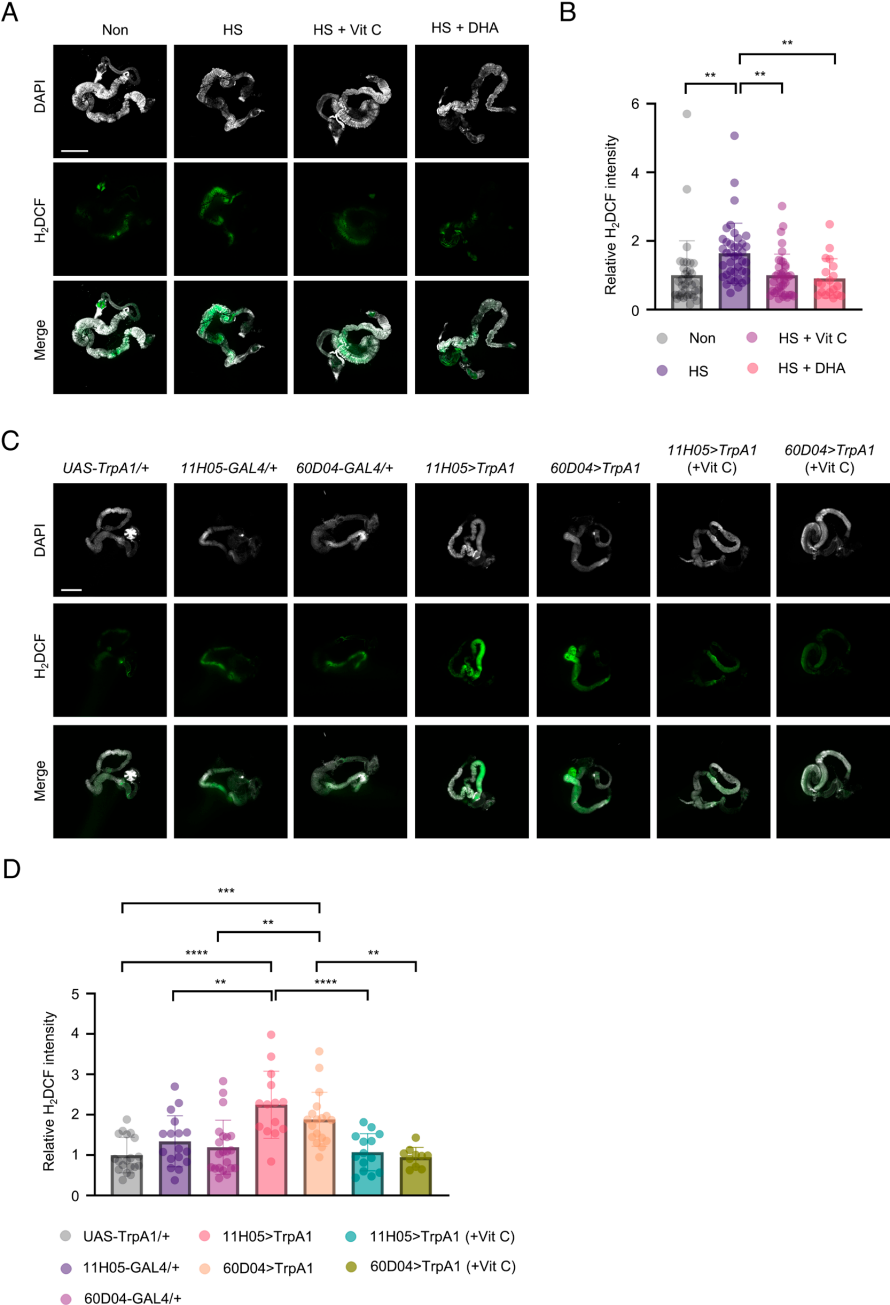
Vitamin C intake reduces the stress-induced increase in ROS levels of the gut. (*A*) Representative images of the gut of heat-stressed flies that had been prefed 40 mM vitamin C, 40 mM DHA, or none for 4 d, stained with DAPI (gray) and H_2_DCF (green). (*B*) Quantifications of H_2_DCF intensities (n = 19 to 40). (*C*) Representative images of the gut of sleep-deprived male flies that had been prefed 10 mM vitamin C or none for 2 d, stained with DAPI (gray) and H_2_DCF (green) on the 7th day. (*D*) Quantifications of H_2_DCF intensities (n = 10 to 21). Data are presented as mean ± SD One-way ANOVA with Tukey’s post hoc test is used. **P < 0.01; ***P < 0.001; ****P < 0.0001. (Scale bar, 500 μm.)

We next investigated the effects of heat stress on the physiology of the internal organs in flies. Using 2’,7’-dichlorodihydrofluorescein diacetate (H_2_DCFDA, a cell-permeable redox-sensitive dye) (60), we measured ROS levels in *Drosophila*. We found that heat stress increased ROS levels significantly in the gut (Fig. 3 *A* and *B*).

Having shown that the heat-induced preference for vitamin C was reduced by prefeeding these flies with vitamin C or DHA, we next sought to determine whether prefeeding them with vitamin C ameliorates the oxidative stress caused by heat stress. We found that ROS levels in the gut of heat-stressed flies that had been prefed vitamin C decreased significantly (Fig. 3 *A* and *B*). Because DHA appeared to be converted to vitamin C (33–35), we investigated the possibility that prefeeding flies with DHA also helps reduce ROS levels. Indeed, ROS levels in the gut of heat-stressed flies that had been prefed supplemental DHA decreased substantially (Fig. 3 *A* and *B*). We also measured ROS levels in the ovary and the brain of heat-stressed flies. While ROS levels in the ovary increased significantly, those in the brain did not change (*SI Appendix,* Fig. S6 *A*–*D*). Prefeeding with vitamin C or DHA did not significantly reduce ROS levels in the ovary (*SI Appendix,* Fig. S6 *A* and *B*). In the brains of heat-stressed flies, DHA prefeeding led to a reduction in endogenous ROS levels (*SI Appendix,* Fig. S6 *C* and *D*). Additionally, when we measured ROS levels in heat-stressed flies prefed with other antioxidants—ferulic acid, gallic acid, NAC, and vitamin E—we found reduced ROS levels in the gut (*SI Appendix,* Fig. S7 *A*–*D*).

In addition to H_2_DCFDA, we employed another redox-sensitive fluorescent reagent, Amplex Red, which specifically detects hydrogen peroxide (*SI Appendix,* Fig. S8*A*). Amplex Red is a nonfluorescent compound that is oxidized to produce resorufin, a highly fluorescent molecule in the presence of hydrogen peroxide and peroxidase (*SI Appendix,* Fig. S8*A*) (61). Using this assay, we found elevated ROS levels in the gut of heat-stressed flies (*SI Appendix,* Fig. S8*B*). By contrast, prefeeding with vitamin C or DHA resulted in a reduction of ROS levels in the gut (*SI Appendix,* Fig. S8*B*). Although heat stress elevated ROS levels in the ovary, prefeeding vitamin C or DHA did not result in a significant reduction (*SI Appendix,* Fig. S8*C*). The ROS level in the brain was not altered by heat stress (*SI Appendix,* Fig. S8*D*). We also assessed gut ROS levels in sleep-deprived flies using H_2_DCFDA staining and confirmed increased ROS levels, which were similarly attenuated by vitamin C prefeeding (Fig. 3 *C* and *D*). Taken together, these findings demonstrate that prefeeding with vitamin C or DHA effectively reduces gut ROS levels, and this reduction is associated with a diminished preference for vitamin C in heat-stressed flies pretreated with vitamin C or DHA.

### The Intake of Vitamin C or DHA Alleviates Gut Leakage and Extends the Survival of Flies Exposed to Chronic Heat Stress.

**Fig. 4.**
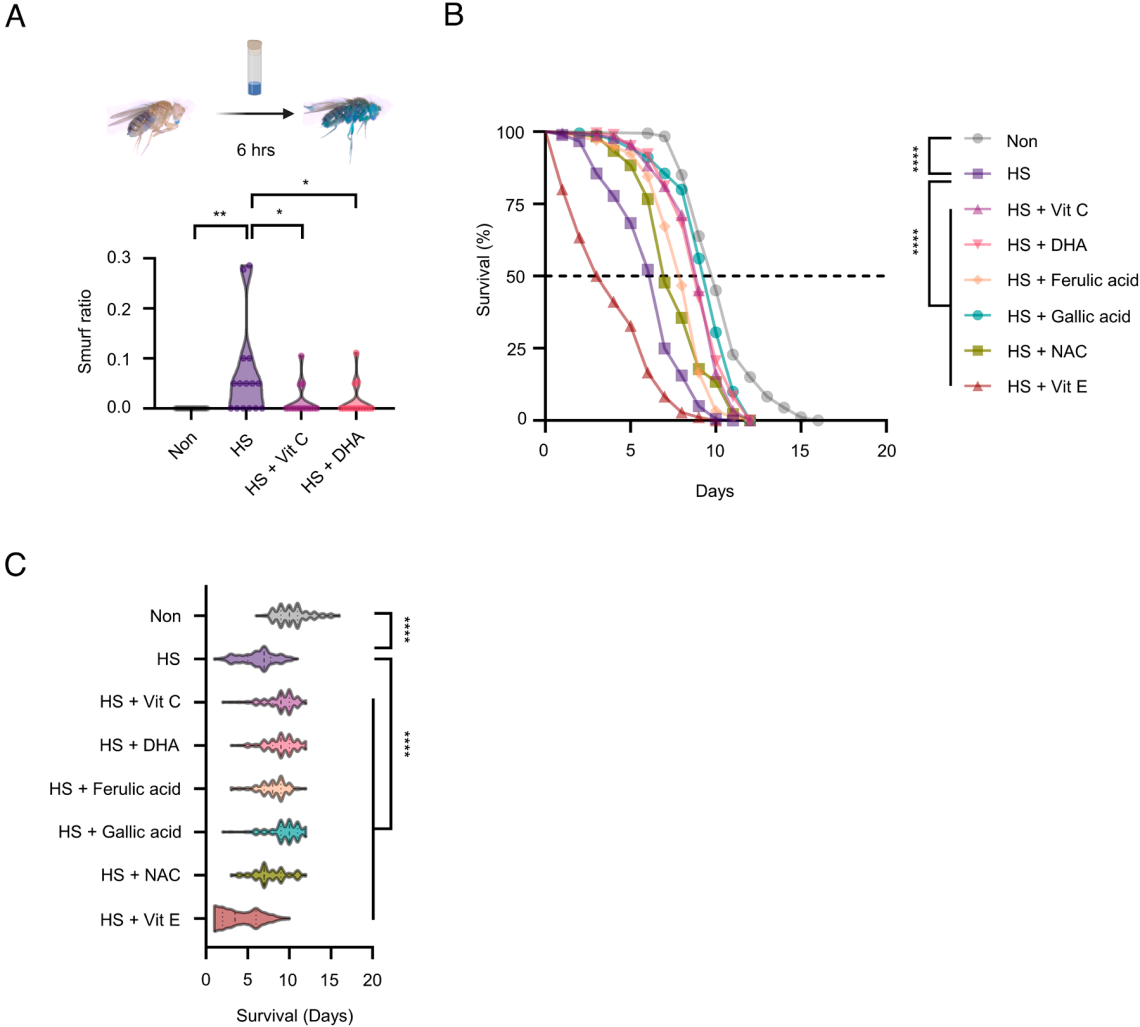
The intake of vitamin C or DHA alleviates gut leakage and extends the survival of flies exposed to chronic heat stress. (*A*) Proportions of Smurf flies that received heat shock, heat shock with postfeeding of 2 mM vitamin C or DHA, or none for 3 d (n = 15). Flies were subjected to a 30 min heat shock once daily for 4 consecutive days. (*B*) Survival curves of heat-stressed flies and those with postfeeding of the denoted antioxidant at 2 mM or none (n = 180). (*C*) Individual plots of (*B*) (n = 180). Flies were subjected to a 30 min heat shock once daily throughout the course of the survival assay. Data are presented as violin plots representing the full distribution of the data for panels *A* and *C*; no error bars are included. Statistical analyses were performed as follows: one-way ANOVA with Tukey’s post hoc test for panel *A*; Kaplan–Meier with log-rank test (Mantel–Cox) for panel *B*; and Welch’s ANOVA with Dunnett’s T3’s post hoc test for panel *C*. *P < 0.05; **P < 0.01; ****P < 0.0001.

Having shown that the increase in ROS levels of the gut caused by heat stress was moderated by feeding of antioxidants, we sought to determine whether the organismal consequences of heat stress would be ameliorated by feeding of antioxidants. Previous works reported that chronic heat exposure caused gut leakages and shortened survival in animals (62, 63). Indeed, we found that heat stress resulted in leakages in the gut of flies using the “Smurf” assay (64), in which the spread of ingested food dye outside of the gut was observed (64). In contrast, the proportion of heat-stressed flies that had the Smurf phenotype was significantly reduced by the consumption of vitamin C or DHA (Fig. 4*A*).

We next inquired whether the behavioral preferences for antioxidants in the heat-stress environment would provide substantial benefits to their well-being. To this end, we determined the survival of flies that had received heat shock for 30 min every day. We first verified that chronic heat stress decreased the survival of flies (Fig. 4 *B* and *C*), as the previous work reported (63). Interestingly, supplementing vitamin C or DHA in food significantly increased the percentage of survival in heat-stressed flies (Fig. 4 *B* and *C*). In addition to vitamin C, we fed heat-stressed flies with other antioxidants, and all tested antioxidants except for vitamin E improved the survival of flies that received chronic heat stress (Fig. 4 *B* and *C*). Together, we showed that the consumption of vitamin C or DHA improved gut damage and increased survival at the organism level, implicating the advantage of antioxidant preference behavior in heat-stressed flies.

### Heat-Induced Vitamin C Preference Occurs Independently of Known Peripheral Receptors.

**Fig. 5.**
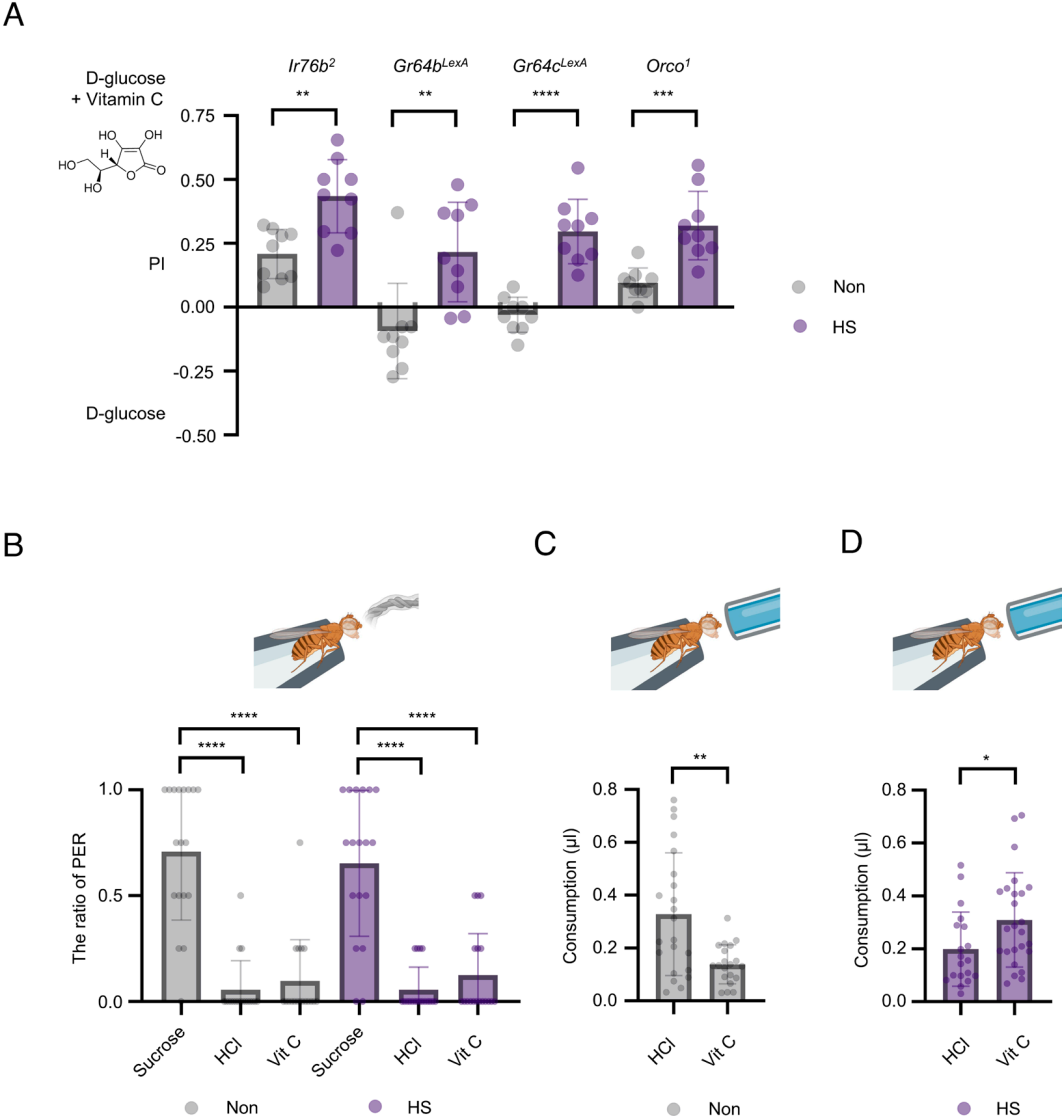
Heat-induced preference for vitamin C is independent of taste and olfactory inputs. (*A*) Two-choice preferences of taste receptor (*Ir76b^2^*, *Gr64b^LexA^, Gr64c^LexA^*) mutants and odorant receptor (*Orco^1^*) mutant flies after exposure to heat stress (n = 9). *Ir76b^2^* and *Gr64b^LexA^* flies were subjected to a 30 min heat shock, and *Gr64c^LexA^* and *Orco^1^* were subjected to a 45 min heat shock. (*B*) PER response of heat-stressed flies to 1 M sucrose, HCl (pH 3.2 to 3.5), and 2 mM vitamin C (n =18). Flies received heat shock for 30 min before conducting the PER assay. (*C*) The intake amount of HCl (pH 3.2 to 3.5) and 2 mM vitamin C by control flies (n = 20 to 22). (*D*) The intake amount of HCl (pH 3.2 to 3.5) and 2 mM vitamin C by heat-stressed flies, which had been subjected to a 30 min heat shock before the MAFE assay (n = 19 to 25). Data are presented as mean ± SD Statistical analyses were performed as follows: unpaired two-tailed *t* test for panels *A*, *C*, and *D*; Welch’s ANOVA with Dunnett’s T3’s post hoc test for panel *B*. *P < 0.05; **P < 0.01; ***P < 0.001; ****P < 0.0001.

In animals, external sensory stimuli are important for making feeding decisions (1, 2). In *Drosophila*, ionotropic receptors, including *Ir76b,* and gustatory receptors, including *Gr64b* and *Gr64c,* are involved in the detection of vitamin C (65). In addition, *Ir76b* also mediates acid sensing, which plays a role in oviposition behavior (66). Hence, we investigated the possibility that these receptors are required for the heat-induced preference for vitamin C. We found that *Ir76b, Gr64b,* or *Gr64c* mutant flies still demonstrated a preference for vitamin C after exposure to heat stress (Fig. 5*A*).

Antioxidants are also detected through the *Orco* receptor in the olfactory system of the fly (67). We investigated the possibility that *Orco* is involved in the heat-induced preference for antioxidants. We found, however, that *Orco^1^* mutant flies exposed to heat stress were still attracted to vitamin C (Fig. 5*A*). This suggests that the known chemosensory receptors in the taste and olfactory systems are not required for the heat-induced vitamin C preference.

We further sought to determine whether the taste responsiveness of flies to vitamin C changed following exposure to heat shock. To this end, we used the PER assay (36) in which each fly was subjected to a wetted wick that measured the taste sensitivity to a solution containing 2 mM vitamin C. We found that the taste responses of heat-stressed flies to 2 mM vitamin C, HCl (acidity control, pH 3.2 to 3.5), or 1 M sucrose were indistinguishable from those of control flies to the same stimuli (Fig. 5*B*). This is consistent with the abovementioned finding that the stress-induced preference for antioxidants may not be mediated by the peripheral chemosensory system.

While the preingestive taste system plays a key role in initiating feeding, postingestive mechanisms may contribute to the detection and response to the therapeutic value of antioxidants. To determine whether heat-stressed flies preferentially consume 2 mM vitamin C (pH 3.2 to 3.5) over the acidity control—HCl, we employed the MAFE assay, which quantifies the food intake of individually tethered flies (37). Interestingly, heat-stressed flies consumed more vitamin C than HCl, whereas control flies showed the opposite preference, consuming more HCl than vitamin C (Fig. 5 *C* and *D*). These results suggest that, under heat stress, the detection and preference for vitamin C may be mediated by a postingestive mechanism rather than the peripheral taste system.

## Discussion

In the wild, animals have to utilize limited resources as carefully as possible to ensure survival and propagation. This promotes the development of specific ingestive behaviors, such as self-medication (12, 13, 15, 16). Self-medication behavior has been referred to as a circumstance related to infection (12, 13, 15, 16). A recent study, however, has expanded the scope to include a pathological condition where flies bearing tumors become attracted to an antitumorigenic compound (aristolochic acid) (68). Another interesting observation is that fruit flies exposed to very low ambient temperatures exhibit a strong preference for plants containing polyunsaturated fatty acids, which may help maintain membrane fluidity and improve their survival in cold environments (69). In this study, we investigated how the feeding behavior of flies is significantly altered after being exposed to oxidative stress instigated by heat shock and sleep deprivation. This observation aligns with prior evidence indicating that oxidative stress can elicit self-medication behavior (14, 17). We found that stressed flies utilize vitamin C and other antioxidants to effectively cope with increased ROS levels in the gut (Figs. 1 and 3). This behavior was observed in both males and females; however, males were more sensitive to heat shock than females, consistent with previous reports (*SI Appendix,* Fig. S1 *B* and *C*) (70, 71). As such, males would respond promptly to the early phase of heat shock but may become vulnerable, impairing their ability to make an appropriate food choice as the heat stress persists (*SI Appendix,* Fig. S1*C*). It would be advantageous for animals to identify and consume exogenous antioxidants to rapidly respond to acute oxidative stress and compensate for the cost of synthesizing new substances (17).

Internal vitamin C levels appear to correlate with flies’ heat-induced preference for vitamin C. We observed that vitamin C levels in the hemolymph of flies decreased following heat shock (Fig. 2*F*). It was known that vitamin C levels also decreased in some livestock during heat stress (72). When flies were fed with vitamin C before the exposure to heat stress, they showed a reduced preference for vitamin C (Fig. 2*A*). This pattern was also observed in flies that were prefed with DHA, which can be converted into vitamin C (Fig. 2*E*) (33–35). Similarly, it has been reported that rats displayed an increased preference for vitamin C when they had been deprived of this nutrient (73). We propose that the preference for vitamin C may serve as an additional mechanism for replenishing this micronutrient during periods of oxidative stress. Indeed, stress-induced increases in gut ROS levels were reduced by prefeeding vitamin C or DHA (Fig. 3 *A* and *B*). Furthermore, the dysfunctional intestinal barrier and shortened survival of flies that had been exposed to chronic heat stress were alleviated by feeding vitamin C or DHA (Fig. 4). This mechanism may be conserved in other animals and might be used by exhausted animals.

There are several directions for future investigation. First, because our behavioral assays were conducted under acute heat-stress conditions, we could not confirm whether the intake of vitamin C in the choice assay truly had a *medicative* effect. The short duration of both heat stress exposure and behavioral tests may not fully reflect the fly’s physiological state immediately. To this end, we examined the long-term effects of vitamin C and found that it restored gut integrity and improved the survival of stressed flies (Fig. 4). We propose that these beneficial outcomes may underlie the observed preference for vitamin C under stress. Second, although some nonpreferred antioxidants also reduced ROS levels in the gut (*SI Appendix,* Fig. S7), they did not elicit a preference behavior (Fig. 1*B*). This could be due to suboptimal assay conditions for those antioxidants or a difference in how their chemosensory preference is developed (32, 74–76). Given that *Drosophila* may detect different antioxidants through specialized sensors located peripherally or internally, feeding behavior is likely selectively modulated depending on the type of antioxidant encountered. These findings underscore that, even within the general class of antioxidants, individual chemicals elicit different behavioral responses in flies (Fig. 1*B*). In nature, fruit flies are known to primarily consume fruits, which are rich in antioxidants such as vitamin C, ferulic acid, and gallic acid (77–80). In contrast, vitamin E is present only at very low levels in most fruits (77). NAC is primarily found in a narrow range, such as allium plants (e.g., onions and garlic) (81), and is not broadly distributed in fruits. This nutritional landscape suggests that fruit flies may have evolved selective sensitivity to antioxidants that are distributed across a wide variety of fruits, making them easily accessible, an aspect reminiscent of the observed dietary preference. Third, it remains unclear how flies sense the properties of different antioxidants. Notably, adjusting the pH of each antioxidant abolished the observed preference (*SI Appendix*, Fig. S4*B*). Previous studies have demonstrated that the chemical form, stability, and antioxidant capacity of compounds can be significantly influenced by pH (46–48, 82–87). Therefore, it is possible that pH-induced changes in these properties may have affected how flies perceive and respond to specific antioxidants.

The detailed mechanism underlying heat-induced preference for vitamin C remains to be elucidated. From an external sensory perspective, it was reported that infected tiger moth caterpillars can alter their taste responses to plant toxins (15). The external sensory system is critical for nutrient selection, and fruit flies are known to detect antioxidants via both olfactory and gustatory systems. Specifically, antioxidant detection has been linked to the olfactory receptor; Orco, as well as taste receptors such as Ir76b, Gr64b, and Gr64c receptors (65, 67). However, in this study, neither of these external sensors appears to be involved in sensing vitamin C under heat-stress conditions (Fig. 5*A*). Heat-stressed flies displayed similar taste responses to vitamin C as control flies, as assessed by the PER assay (Fig. 5*B*). By contrast, only heat-stressed flies consumed significantly more amounts of vitamin C compared to an acidity control, as shown in the MAFE assay (Fig. 5 *C* and *D*). In previous studies, the deprivation-induced preferences for particular macronutrients were mediated independently of taste-related inputs (3, 4, 6). Similarly, the heat-induced preference for vitamin C observed here appears to be mediated independently of the peripheral chemosensory inputs, although the underlying mechanism remains unknown. While our study has not fully elucidated the basis of this self-medication behavior, our findings suggest that it may be regulated by a postingestive mechanism. Future research should aim to identify and characterize the interoceptive sensors that detect essential micronutrients like antioxidants and mediate these adaptive behavioral responses.

## Materials and Methods

Experiments were conducted using *Drosophila melanogaster*, mainly with *w^1118^* mated females unless otherwise noted. Flies were reared on cornmeal-based food at 25 °C under a 12 h:12 h light/dark cycle. Nutrient preference and intake were assessed using dye-based two-choice assays (38), BARCODE-based (39, 40), and absorbance-based (41, 43) quantification methods. These were quantified using qPCR for BARCODE assays and a microplate reader for absorbance-based assays. Hemolymph was extracted using spin columns (88), and the concentrations of sugar, protein, and lipid in hemolymph were measured via anthrone, Bradford, and sulfo-phospho-vanillin (SPV) assays, respectively (52, 56). The antioxidant capacity of the hemolymph and chemicals was measured using a total antioxidant capacity assay kit. Relative vitamin C levels were measured via LC–MS. Oxidative stress and antioxidant effects were evaluated using H_2_DCFDA staining (89) and the Amplex Red assay (90). Additional assays included sleep monitoring using the *Drosophila* Activity Monitor (DAM) system to validate genetically induced sleep deprivation (44), the Smurf assay to assess gut integrity (64), antioxidant-mediated survival analysis (63), and behavioral responses to nutrients using PER (36, 91) and manual feeding (MAFE) assays (37). Detailed experimental protocols, reagent information, quantification, and statistical analysis are provided in the *SI Appendix*, *Supplementary Materials and Methods*.

## Supplementary Material

Appendix 01 (PDF)

## Data Availability

All study data are included in the article and/or *SI Appendix*.
