# Supplementary material for: Stress-induced preference for antioxidants by Drosophila
Source: Proc Natl Acad Sci U S A. 2025 Sep 18;122(38):e2512852122. doi: 10.1073/pnas.2512852122 (PMC12478143; doi:10.1073/pnas.2512852122)
Supplement: Supplementary file 1 — Appendix 01 (PDF) [file pnas.2512852122.sapp.pdf]

PNAS

Supporting Information for

Stress-induced preference for antioxidants by *Drosophila*

Gayoung Hwang[1*], Dae-Wook Yang[1*], Terezia Klaudia Geisseova[2], Hae A Kim[3], Yangkyun Oh[4], and Greg S. B. Suh[1†]

[1] Department of Biological Sciences, Korea Advanced Institute of Science and Technology (KAIST), Daejeon 34141, Republic of Korea

[2] Department of Biological Sciences, Ulsan National Institute of Science and Technology (UNIST), Ulsan 44919, Republic of Korea

[3] KAIST Analysis Center for Research Advancement (KARA), Korea Advanced Institute of Science and Technology (KAIST), Daejeon 34141, Republic of Korea

[4] Department of Life Science, College of Natural Sciences, Ewha Womans University, Seoul 03760, Republic of Korea

*These authors contributed equally to this work.

†Corresponding author: Greg S. B. Suh

Email: seongbaesuh@kaist.ac.kr

This PDF file includes:

Supplementary Materials and Methods

SI References

Figures S1 to S8

Supplementary Materials and Methods

Fly husbandry

w^{1118} and *Canton-S* flies were used as wild-type. *$Ir76b^2$* (BDSC 51310), *$Orco^1$* (BDSC 23129), *$Gr64b^{LexA}$* (BDSC 93442), *$Gr64c^{LexA}$* (BDSC 93443), *11H05-GAL4* (BDSC 45016), *60D04-GAL4* (BDSC 45356), and *UAS-TrpA1* (BDSC 26263) were obtained from the Bloomington *Drosophila* Stock Center (BDSC) and Dr. Youngseok Lee's laboratory. All flies were reared on cornmeal-based standard food (9 L tap water, 80 g agar, 671 g corn meal, 92 g soy flour, 159 g yeast, 700 ml starch syrup, 50 ml of 30% tegosept (in Ethanol), and 45 ml propionic acid for 10 L of normal fly food) in the 25 ºC incubator with the 12 h:12 h light and dark cycle. Experiments were conducted with *w^{1118}* mated female flies unless mentioned otherwise.

Chemicals

Chemicals were dissolved in each solvent as follows: L-ascorbic acid (Vitamin C) (A5960, Sigma), N-Acetyl-L-cysteine (NAC) (A7250, Sigma), in Distilled water (DW); L-Dehydroascorbic acid (DHA) (261556, Sigma), *trans*-Ferulic acid (Ferulic acid) (128708, Sigma) in DMSO; Gallic acid (C41086, Alfa Aesar) in 75% Ethanol (EtOH); and (±)-α-Tocopherol (Vitamin E) (T3251, Sigma) in 100% EtOH.

Two-choice assay

The two-choice assay was conducted as previously described with few modifications (1). Briefly, approximately 30 flies at 2-3 days old were collected under CO_2 anesthesia and transferred to fresh food after 2-3 days. On the test day, 6-8 days old flies were starved for 3 h in an empty vial with 2 ml of DW-soaked Kimwipe in a 25 ºC incubator. Flies were either given heat shock at 38 °C during the final 1 h or placed in a 25 ºC incubator. Heat shock was delivered for 1 h unless mentioned otherwise. Flies were then anesthetized on ice and introduced to a 72-well plate (438733, Thermo Fisher Scientific). The two-choice assay was conducted for 2 h (ZT 6-8) in a 25 ºC incubator in the dark, and the preference index (PI) was calculated as previously described (2). For the time-course experiment, the two-choice assay was performed for different durations (10, 20, 30, and 120 min).

For pre-feeding experiments, flies were collected, recovered for 1 day, and reared on supplemented food for 4 days. The supplemented food was produced by adding a denoted chemical to the standard food. A denoted chemical was dissolved in each solvent as mentioned above. Fresh-supplemented foods were provided every other day. The two-choice assay was performed using the same procedure described above.

To prepare foods in the two-choice arena, 1% aqueous agar (A5306, Sigma) was mixed with 100 mM D-glucose (G8270, Sigma) and a denoted chemical. The two types of food were colored with different dyes (3% red food dye and 1.5% green food dye, McCormick). Switching the color associated with each food was performed. Most behavior assays were inspected by two independent experimenters to eliminate personal bias.

To prepare foods containing chemicals (Vitamin C, NAC, Ferulic acid, Gallic acid, Vitamin E, and DHA), those chemicals were dissolved in each solvent as mentioned above. To prepare an acidic food, 1 M HCl (320331, Sigma) was used to adjust pH 3.2-3.5, which is close to the pH of 2 mM vitamin C. In the case of making other acidic foods, Citric acid

(251275, Sigma) and Glycolic acid (798053, Sigma) were dissolved in DW, and diluted to 2 mM concentration (about pH 2.98, pH 3.23). Acetic acid (A/0400/PB15, Fisher Chemical) was diluted to 2 mM (about pH 3.11). To prepare food containing acidified Vitamin E (about pH 3.37) or neutralized antioxidants (Vitamin C, Ferulic acid, Gallic acid, and NAC) (about pH 6-7), we used 1 M HCl or 2 N NaOH (SS255-1, Fisher Scientific) to adjust their pH.

BARCODE-based measurement of food intake

To measure the consumption of two different food sources, the BARCODE-based assay was performed as previously reported (3, 4). DNA oligomers, including qPCR primers, were synthesized by Macrogen Inc. (South Korea). The sequences of them were referred to in a previous report (4).

The two-choice assay was performed using the same procedure described above. Vitamin C-free food was labeled with DNA oligomer 1, and vitamin C-containing food was labeled with DNA oligomer 2. After the assay was completed, thirty female flies were collected in an EP tube. To remove the oligomers adhering to the surface of the flies, we performed a four-step wash as previously described with few modifications (3, 4): 10 min wash with 5% detergent (Z742914**,** Sigma); 5 min wash with DW; 2 min wash with 30% bleach; and 5 min wash with DW. Washed flies were homogenized using Pellet Pestle® Motor Kontes in 800 µl of lysis buffer (10 mM Tris-HCl pH 8.2, 1 mM EDTA, and 1 mM NaCl) with 1.6 µl of 20 mg/ml proteinase K (AM2546, Thermo Fisher Scientific). The lysates were incubated at 37 °C for 30 min, followed by proteinase K inactivation at 95 °C for 5 min. The samples were centrifuged at 15,000 rpm for 20 min at room temperature, and then supernatants were collected for further analyses.

2 µl of the supernatants were used in a 20 µl-reaction of qPCR experiment, which was performed using SYBR green PCR master mix (4367659, Thermo Fisher Scientific) and a CFX96 Touch Real-Time PCR Detection System (Bio-Rad) with a setting of 40 cycles per run (T_m = 60 °C), and obtain the cycle threshold (Ct) values of supernatants. For quantifying food intake, the standard curves of each oligomer were generated. Ct values of the *Cyclophilin 1* (*Cyp1*) gene in flies were used as an indicator of extraction efficiency. To determine the untargeted pairing in amplification, we used non-oligomer samples as negative controls. Only samples with Ct values between the positive (targeted pairing) and negative controls were used for quantification.

Absorbance-based measurement of food intake

To evaluate the consumption of two different foods, we performed a two-choice assay and measured the absorbance of the sample of flies that consumed dyes in the foods. The two-choice assay was performed using the same procedure described above. The two types of food were colored with different dyes: 1% red dye (Allura Red AC, 458848, Sigma) and 3% blue dye (Brilliant Blue FCF, 027-12842, Wako). These dyes exhibit minimal spectral cross-talk at 504 nm and 630 nm (5, 6). To calculate the net absorbance, control plates without added dye were also prepared.

After the assay was completed, 30 female flies were transferred to an EP tube and moved into a -80 ºC deep freezer. Flies were ground using Pellet Pestle® Motor Kontes in lysis buffer (1% Triton X-100 in PBS). Ground samples were vortexed and

centrifuged at 15,000 rpm for 20 min at room temperature. Supernatants were collected, and loaded into the 96-well clear Costar Assay plate (9017, CORNING).

The absorbance wavelengths of the supernatant samples were measured at 504 nm (Allura Red) and 630 nm (Brilliant Blue) using a multi-functional microplate reader (SPARK, Tecan) with the SPARK CONTROL program in the KAIST Bio Core Center. The absorbance of the supernatant samples incubated in the dye-free plate was subtracted from those incubated in the dye-containing plate. To determine food intake (FI), the standard curves for 1% red dye and 3% blue dye were generated (5, 7). The preference index (PI) value was calculated as $(FI_{504\ nm} - FI_{630\ nm})/(FI_{504\ nm} + FI_{630\ nm})$, referred to in a previous report with few modifications (7).

Collection of flies' hemolymph

Approximately 80 female flies at 6-8 days old were collected and transferred to fresh foods in a 25 ºC incubator for 2 h before receiving heat shock for 1 h in a 38 ºC incubator. The collection procedure for flies' hemolymph was modified from the previous study (8). Flies were transferred to zymo-spin III CG columns (C1006-50-G, ZYMO RESEARCH) and covered with 2 mm glass beads (KA.UB33-21B, Korea Ace Scientific). The columns were placed in EP tubes and centrifuged at 14,000 rpm for 30 min at 4 °C, twice. The supernatants were transferred to new EP tubes and centrifuged again at 14,000 rpm for 5 min at 4 °C.

Measurement of the antioxidant capacity

The antioxidant capacities of vitamin C, DHA, and hemolymph samples were measured using an OxiTec^{TM} total antioxidant capacity assay kit (BO-TAC-200, BIOMAX) with a few modifications. 100 μl of 2 mM vitamin C or DHA or 2 μl of hemolymph (with 98 μl of DW) was mixed with 100 μl of copper reagent and 100 μl of reaction buffer. Samples were loaded into the 96-well clear Costar Assay plate (9017, CORNING) and incubated for 30 min at room temperature. The absorbance at 450 nm was measured using a multi-plate reader (SpectraMax ID3, Molecular Devices) with SoftMax Pro7.1 program or a multi-functional microplate reader (SPARK, Tecan) with the SPARK CONTROL program in the KAIST Bio Core Center. To quantify the antioxidant capacities of vitamin C, DHA, and hemolymph samples, the standard curve using trolox was generated.

Anthrone assay

To measure the concentration of sugar in hemolymph, anthrone assay was referred to in previous reports with few modifications (9). 1 μl of hemolymph and glucose standard samples were mixed with 1 ml of anthrone reagent; 2 mg/ml anthrone (319899, Sigma) in 72.375% sulfuric acid (258105, Sigma) (10, 11). The sample mixture was incubated at 90 ºC for 10 min using a heat block and incubated for 5 min at room temperature. Samples were loaded into the 96-well clear Costar Assay plate (9017, CORNING). The absorbance wavelengths were measured at 625 nm using a multi-functional microplate reader (SPARK, Tecan) with the SPARK CONTROL program in the KAIST Bio Core Center. To quantify the concentration of sugar in hemolymph, the standard curve using glucose was generated.

Bradford assay

The concentration of protein in hemolymph was measured using Bio-Rad protein assay dye reagent concentrate (500-0006, Bio-Rad), following the manufacturer's guideline and a report with few modifications (9). 2 μl of hemolymph was mixed with 998 μl of Bradford assay solution (20%). 2 mg/ml Bovine Serum Albumin (BSA) (BB015, Bio-solution) was used as a sample to generate the standard curve. Samples were loaded into cuvettes (2712120, Ratiolab) and incubated for 5 min at room temperature. The absorbance wavelengths were measured at 595 nm using a Vis Spectrophotometer (OPTIZEN POP-V, Keen Innovative Solutions).

Sulfo-phospho-vanillin (SPV) assay

To measure the concentration of lipids in hemolymph, the Sulfo-phospho-vanillin (SPV) assay was referred to previous reports with few modifications (9, 12). 4 μl of hemolymph and standard samples; soybean oil (7656-1405, DAEJUNG) in EtOH were evaporated at 90 ºC for 10 min using a heat block. 40 μl of sulfuric acid (258105, Sigma) was added, and samples were incubated at 90 ºC for 10 min using a heat block. After 5 min of incubation at room temperature, 960 μl of a solution of the phosphoric acid reagent (1.00573, Sigma) with vanillin (8.18718, Sigma) (SPV) or phosphoric acid reagent without vanillin (SP) was added (13). Samples were loaded into the 96-well clear Costar Assay plate (9017, CORNING) and incubated for 10 min at room temperature. The absorbance wavelengths were measured at 525 nm using a multi-functional microplate reader (SPARK, Tecan) with the SPARK CONTROL program in the KAIST Bio Core Center. To eliminate interference from the coloration of non-target substances during acid digestion, we calculated the net absorbance of the hemolymph samples (13). To quantify the concentration of lipids in hemolymph, the standard curve using soybean oil was generated.

LC-MS analysis

The relative levels of vitamin C in flies' hemolymph or neutralized vitamin C were measured using UPLC/Q-TOF Mass Spectrometry (XEVO G2-XS Q-Tof, Waters Corporation) in the KAIST Analysis Center for Research Advancement (KARA). 10 μl of hemolymph (with 10% DW) or 1 μl of neutralized vitamin C samples were injected into a reverse-phase column (186002350, ACQUITY UPLC BEH, C18, 2.1 x 50 mm, 1.7 μm particle size, Waters Corporation) and separated at a flow rate of 0.3 ml/min at 25 ºC. The mobile phase consisted of two buffers: 0.1% formic acid in DW and 0.1% formic acid in ACN. The electrospray ionization (ESI) was used in negative ion mode. The capillary voltage was 2.5 kV. We optimized the gradient conditions for the chromatography: 0-3 min at a linear gradient from 10% to 15% B, 3-3.1 min at a linear increase to 100% B, 3.1-5 min at 100% B, and re-equilibration at 10% B for 2 min. Vitamin C dissolved in hemolymph or DW was used to determine the retention time, m/z values, and MS/MS fragment pattern. m/z value for vitamin C is 175.025. Data processing was performed using software (TargetLynx, Waters).

For the preparation of neutralized vitamin C, 2 mM vitamin C samples were neutralized using 2 N NaOH and transferred into a 25 ºC incubator for 3 hours. Once incubation was

completed, the samples were diluted, and their vitamin C levels were measured using LC-MS.

H_2DCFDA staining

ROS levels in the organs (gut, ovary, and brain) were measured using H_2DCFDA (D399, Invitrogen) as previously described with minor modifications (14). H_2DCFDA was dissolved in anhydrous DMSO (276855, Sigma) to make an aliquot of 10 mM H_2DCFDA stock solution. The stock solution was prepared immediately before each experiment. The pre-feeding scheme was the same procedure as the two-choice assay. The organs were dissected in PBS and incubated for 15 min with 10 μM H_2DCFDA and 2 μg/ml DAPI (D9542, Sigma) (in PBS) and washed with PBS for 5 min, three times. For fixation, the organs were incubated with 4% PFA (in PBS) for 5 min and washed with PBS for 5 min once. All staining steps were performed using a rotator (except for the fixation) at room temperature in the dark. Samples were mounted using Vectashield Antifade Mounting Medium (H-1000, Vector Laboratories) and immediately imaged using a confocal microscope (LSM780, ZEISS, 5x lens; gut and ovary, 10x lens; brain) in the BioMedical Research Center (BMRC), KAIST or a confocal microscope (AX R Confocal Microscope System, Nikon, 2x lens). Image processing was performed using software (ZEISS, Zen 2012 black edition, Zen 2.5 blue edition; Nikon, NIS elements AR). To quantify the H_2DCFDA labeling, we used ImageJ software.

Amplex Red assay

For the detection of hydrogen peroxide, we used Amplex^{TM} Red (A12222, Invitrogen) following the manufacturer's instructions and a previous report with minor modifications (15). Amplex Red was dissolved in anhydrous DMSO (276855, Sigma) to make an aliquot of 10 mM stock solution. The pre-feeding scheme was the same procedure as the two-choice assay. The organs (gut, ovary, and brain) were dissected in PBS (5 organs / well) and incubated for 30 min with 5 μM Amplex Red (in PBS) at room temperature. The sample without organs was also incubated with Amplex Red as a background control, and its reading was subtracted from those of the experimental samples. The supernatants were loaded into the 96-well clear Costar Assay plate (9017, CORNING), and the fluorescence intensities were measured at 530/590 nm (Excitation/Emission) wavelength using a multi-functional microplate reader (SPARK, Tecan) with the SPARK CONTROL program in the KAIST Bio Core Center.

Sleep deprivation

Sleep deprivation of flies was performed as previously described with few modifications (16). Briefly, flies carrying either *11H05-GAL4* or *60D04-GAL4* with *UAS-TrpA1*, as well as control flies, were raised at 21 °C until day 3–4 post-eclosion. Approximately 30 male flies were collected per vial. These flies were transferred to a 29 °C incubator and kept there for 7 days to induce sleep deprivation. Some of these flies were pre-fed 10 mM vitamin C for the last 2 days followed by the subsequent experiments. The two-choice assay and H_2DCFDA staining followed the same protocol as above, with the exception that starvation and fixation procedures were omitted (16).

***Drosophila* Activity Monitor (DAM) assay**

To validate whether flies exhibited sleep loss, we used the DAM assay. 3-4 days old male flies were individually placed into a 65 x 5 x 5 mm glass tube. At the end of the tube, food containing 5% sucrose (S0389, Sigma) with 2% agar (214010, Difco) was located. The fly-containing tubes were loaded into *Drosophila* Activity Monitor system boards (DAM2, Trikinetics) with 1 infrared beam sensor and placed in the 29 ºC incubator for 7 days with a 12 h:12 h light:dark cycle. The data of activity bins were collected via DAMSystem308X software (Trikinetics) in 1-minute intervals. The locomotion data were further processed via the DAMFileScan107 (Trikinetics) software, and the quantification of sleep duration was performed via an Excel macro (17). The sleep episode threshold was set to at least 5 minutes of flies' inactivity.

Smurf assay

Smurf assay was performed as previously described with few modifications (18). The treatment scheme was the same as the survival assay, except for treating heat shock for 30 min, once a day, for 4 days. On the last day of heat shock, approximately twenty female flies were transferred to standard food containing 2.5% brilliant blue FCF (027-12842, FUJIFILM Wako Pure Chemical Corporation) and placed in a 25 ºC incubator for 6 hours. The Smurf phenotype was scored when the spread of blue food dye was observed outside the digestive tract of the tested flies. For statistical analysis, the number of Smurf flies was divided by the number of flies that consumed food with blue dye.

Survival assay

Survival assay was performed as previously described with few modifications (19). Briefly, twenty female flies at 6-8 days old were transferred into empty vials and placed in a 25 ºC (non-treated) or 38 ºC incubator for 30 min every day. They were then transferred to foods containing 1% agar with 100 mM D-glucose (non-, heat shock-), or 1% agar with 100 mM D-glucose supplemented with 2 mM of each chemical (heat shock with post-feeding of chemicals). All tested flies were transferred to fresh vials every other day, and the number of dead flies was counted every day. This experiment was conducted until all flies were dead.

Proboscis Extension Response (PER) assay

The PER assay was performed as previously described with few modifications (20, 21). On the test day, 6-8 day-old female flies were wet-starved for 2 h 30 min. To enhance their responsiveness to sucrose (used as a positive control), flies were subjected to a final 30-minute starvation period in empty vials without food or moisture. For heat shock-treated flies, this period was conducted at 38 °C, while for untreated flies, it was conducted at 25 °C.

In the PER assay, a single fly was restrained in a knife-cut yellow pipette tip using an aspirator, and a Kimwipe wick soaked in the test solution was gently applied to the fly's labellum to assess its gustatory responsiveness. We tested whether the fly responded to DW (negative control) and responded to 1 M sucrose (positive control). Afterward, DW

was applied to the proboscis at least 10 times to induce satiation. Then, the fly was tested for its responsiveness to the test substance (1 M sucrose, 2 mM vitamin C, or HCl; pH 3.2-3.5). To calculate the ratio of PER, we determined how many times the fly responded to the test substance when applied four times.

Manual Feeding (MAFE) assay

The MAFE assay was performed as previously described with few modifications (22). The method of wet-starvation and heat shock treatment was the same procedure as the PER assay. Before performing the MAFE assay, we tested for fly's responsiveness to DW (negative control) and 1 M sucrose (positive control) and applied DW to the labellum at least 10 times to induce satiation.

A liquid substance (vitamin C or HCl; pH 3.2-3.5) in a microcapillary tube (P0549, Sigma) was placed in the fly's proboscis. The amounts of consumption were measured using a Stainless Steel Vernier Caliper by measuring volume changes, with each measurement repeated 10 times. Trials in which flies did not respond within approximately 10 seconds were excluded from the analysis.

Quantification and statistical analysis

Prism 10 (GraphPad) software was used for statistical analysis and representation of the graphical data. Two groups' comparisons were analyzed using unpaired Student t-test (in case of no difference in s.d) or Welch's t-test (in case of significant difference in s.d). Multiple group comparisons were analyzed using one-way ANOVA with Tukey's post hoc test (in case of no difference in s.d) or Welch's ANOVA with Dunnett's T3's post hoc test (in case of significant difference in s.d). Survival percentage was analyzed using Kaplan-Meier with Log-rank test (Mantel-Cox). Significance is defined by the rule of Prism 10 software: *$P < 0.05$; **$P < 0.01$; ***$P < 0.001$; ****$P < 0.0001$. Error bars are presented as mean ± s.d in the graphical data of most experiments; and mean ± s.e.m in the graphical data of sleep profile and sleep quantification.

SI References

1. M. Dus, S. Min, A. C. Keene, G. Y. Lee, G. S. Suh, Taste-independent detection of the caloric content of sugar in Drosophila. *Proc Natl Acad Sci U S A* **108**, 11644–11649 (2011).
2. M. Dus, M. Ai, G. S. Suh, Taste-independent nutrient selection is mediated by a brain-specific Na+/solute co-transporter in Drosophila. *Nature neuroscience* **16**, 526–528 (2013).
3. A. Park, T. Tran, N. S. Atkinson, Monitoring food preference in Drosophila by oligonucleotide tagging. *Proc Natl Acad Sci U S A* **115**, 9020–9025 (2018).
4. X. Gu *et al.*, Sestrin mediates detection of and adaptation to low-leucine diets in Drosophila. *Nature* **608**, 209–216 (2022).
5. G. Wu *et al.*, Opposing GPCR signaling programs protein intake setpoint in Drosophila. *Cell* **187**, 5376–5392 e5317 (2024).
6. A. T. Bisgin, Simultaneous Extraction and Determination of Allura Red (E129) and Brilliant Blue FCF (E133) in Foodstuffs by Column Solid-Phase Spectrophotometry. *J AOAC Int* 10.5740/jaoacint.18-0073 (2018).
7. B. C. Shell, Y. Luo, S. Pletcher, M. Grotewiel, Expansion and application of dye tracers for measuring solid food intake and food preference in Drosophila. *Sci Rep* **11**, 20044 (2021).
8. S. A. Lindsay, S. J. H. Lin, S. A. Wasserman, Short-Form Bomanins Mediate Humoral Immunity in Drosophila. *J Innate Immun* **10**, 306–314 (2018).
9. S. Lebreton, E. Darrouzet, C. Chevrier, Could hosts considered as low quality for egg-laying be considered as high quality for host-feeding? *J Insect Physiol* **55**, 694–699 (2009).
10. A. Moraru *et al.*, Elevated Levels of the Reactive Metabolite Methylglyoxal Recapitulate Progression of Type 2 Diabetes. *Cell Metab* **27**, 926–934 e928 (2018).
11. K. Aditi, A. Singh, M. N. Shakarad, N. Agrawal, Management of altered metabolic activity in Drosophila model of Huntington's disease by curcumin. *Exp Biol Med (Maywood)* **247**, 152–164 (2022).
12. E. Van Handel, Rapid determination of total lipids in mosquitoes. *J Am Mosq Control Assoc* **1**, 302–304 (1985).
13. J. Farinacci, J. Laurent, Critical assessment of the sulfo-phospho-vanillin method to quantify lipids in freeze-dried microalgae. *Journal of Applied Phycology* **35**, 997–1008 (2023).

14. E. Owusu-Ansah, A. Yavari, U. Banerjee, A protocol for in vivo detection of Reactive Oxygen Species. *Protocol Exchange* 10.1038/nprot.2008.23 (2008).
15. J. H. M. Oliveira *et al.*, Catalase protects Aedes aegypti from oxidative stress and increases midgut infection prevalence of Dengue but not Zika. *PLoS Negl Trop Dis* **11**, e0005525 (2017).
16. A. Vaccaro *et al.*, Sleep Loss Can Cause Death through Accumulation of Reactive Oxygen Species in the Gut. *Cell* **181**, 1307–1328 e1315 (2020).
17. C. Pfeiffenberger, B. C. Lear, K. P. Keegan, R. Allada, Processing circadian data collected from the Drosophila Activity Monitoring (DAM) System. *Cold Spring Harb Protoc* **2010**, pdb prot5519 (2010).
18. M. Rera, R. I. Clark, D. W. Walker, Intestinal barrier dysfunction links metabolic and inflammatory markers of aging to death in Drosophila. *Proc Natl Acad Sci U S A* **109**, 21528–21533 (2012).
19. R. Elvira, S. J. Cha, G. M. Noh, K. Kim, J. Han, PERK-Mediated eIF2alpha Phosphorylation Contributes to The Protection of Dopaminergic Neurons from Chronic Heat Stress in Drosophila. *Int J Mol Sci* **21** (2020).
20. T. Shiraiwa, J. R. Carlson, Proboscis extension response (PER) assay in Drosophila. *J Vis Exp* 10.3791/193, 193 (2007).
21. H. K. M. Dweck, G. J. S. Talross, Y. Luo, S. A. M. Ebrahim, J. R. Carlson, Ir56b is an atypical ionotropic receptor that underlies appetitive salt response in Drosophila. *Curr Biol* **32**, 1776–1787 e1774 (2022).
22. W. Qi *et al.*, A quantitative feeding assay in adult Drosophila reveals rapid modulation of food ingestion by its nutritional value. *Mol Brain* **8**, 87 (2015).

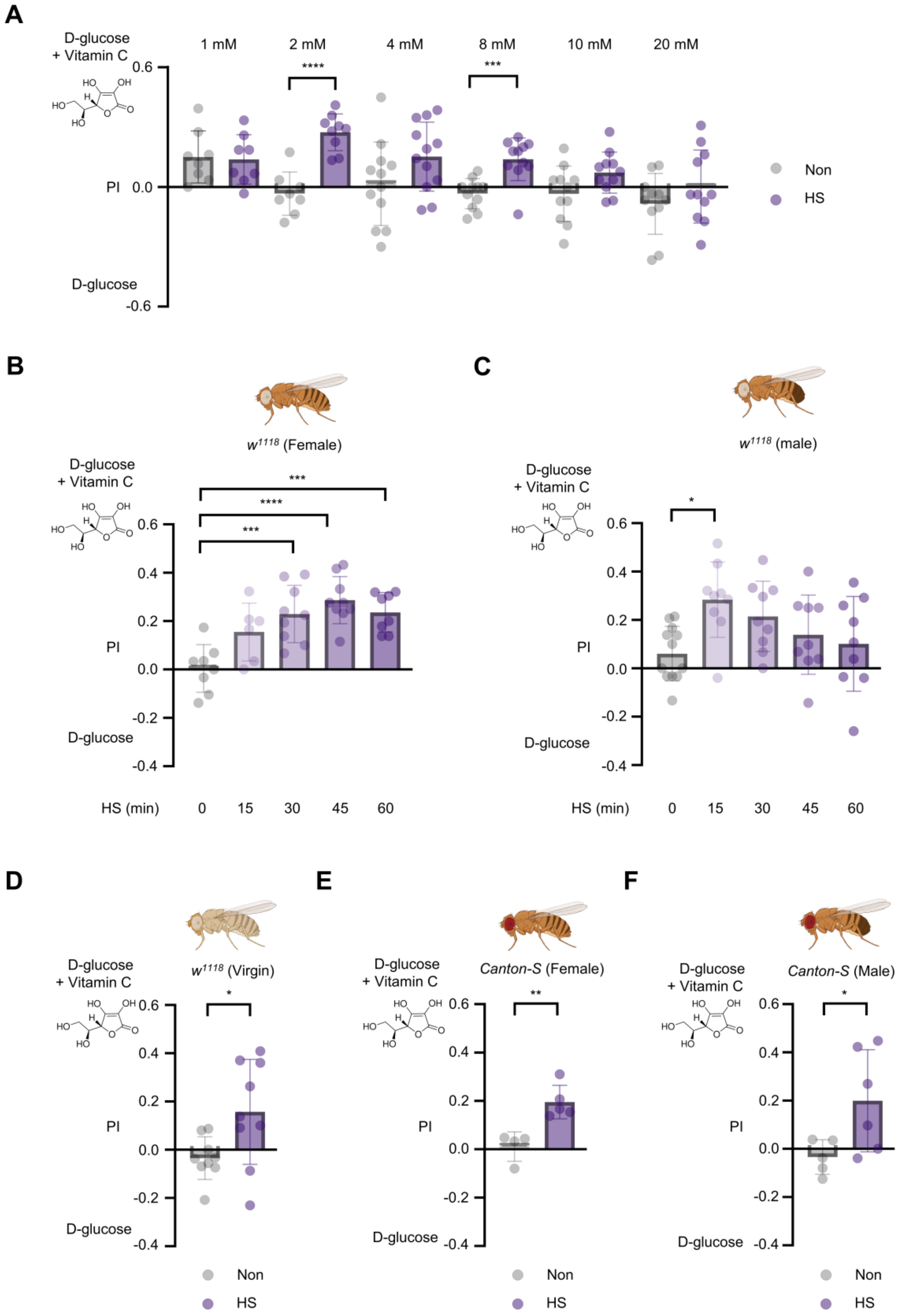
A
D-glucose
+ Vitamin C
1 mM
2 mM
4 mM
8 mM
10 mM
20 mM
****
***
PI
D-glucose
Non
HS
B
w1118 (Female)
***
****
***
HS (min)
0
15
30
45
60
C
w1118 (male)
*
D
w1118 (Virgin)
*
E
Canton-S (Female)
**
F
Canton-S (Male)
*

Figure S1. The preference for vitamin C is influenced by its concentration, the duration of heat shock, sex, and strain.

(A) Two-choice behavioral preferences of heat-stressed flies to different concentrations of vitamin C (1, 2, 4, 8, 10, and 20 mM) (n = 8-12). (B, C) Two-choice preferences of (B) mated females (n = 6-9) and (C) males (n = 9-12) after exposure to different durations of heat stress (0, 15, 30, 45, and 60 minutes). (D-F) Two-choice preferences of (D) w^{1118} virgin flies after receiving a 1-hr heat shock (n = 9), (E) *Canton-S* female flies after receiving a 30-minute heat shock (n = 4-5), and (F) *Canton-S* male flies after receiving a 15-minute heat shock (n = 5-6). Data are presented as mean ± s.d. Statistical analyses were performed as follows: unpaired two-tailed t-test for panels A and D-F; one-way ANOVA with Tukey's post hoc test for panels B and C. *$P < 0.05$; **$P < 0.01$; ***$P < 0.001$; ****$P < 0.0001$.

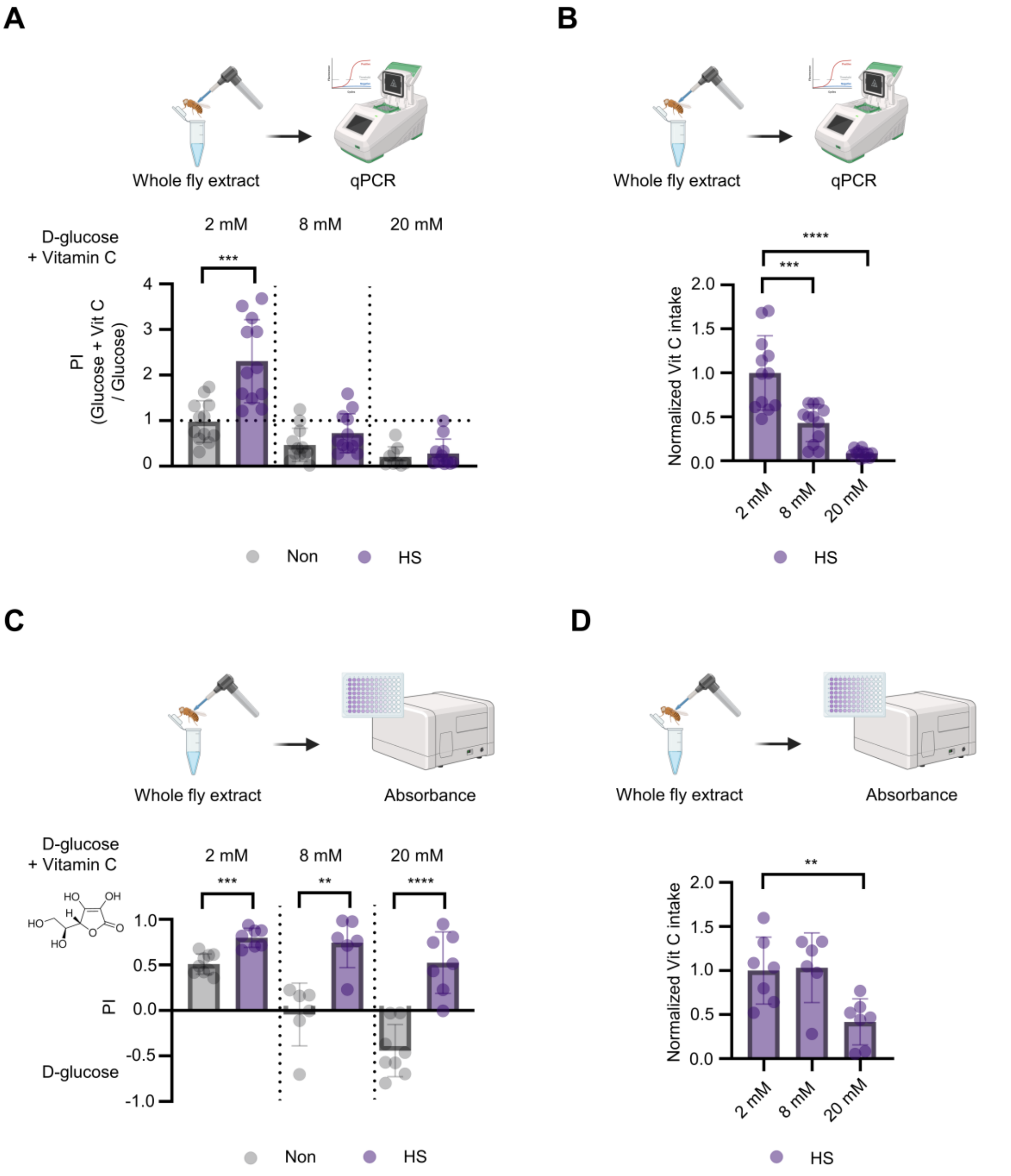

Figure S2. Measurement of vitamin C intake using quantitative feeding assays.
(A, B) Feeding measurement using a BARCODE-based assay. (A) Preferences of heat-stressed flies for food containing different concentrations of vitamin C (2, 8, and 20 mM) (n = 9–12); (B) Normalized intake of vitamin C by heat-stressed flies (n = 11-12). (C, D) Feeding measurement using an absorbance-based assay. (C) Preferences of heat-stressed flies for food containing different concentrations of vitamin C (2, 8, and 20 mM) (n = 6-8); (D) Normalized intake of vitamin C by heat-stressed flies (n = 6-7). Data are presented as mean ± s.d. Unpaired two-tailed t-test is used. $^{**}P < 0.01$; $^{***}P < 0.001$; $^{****}P < 0.0001$.

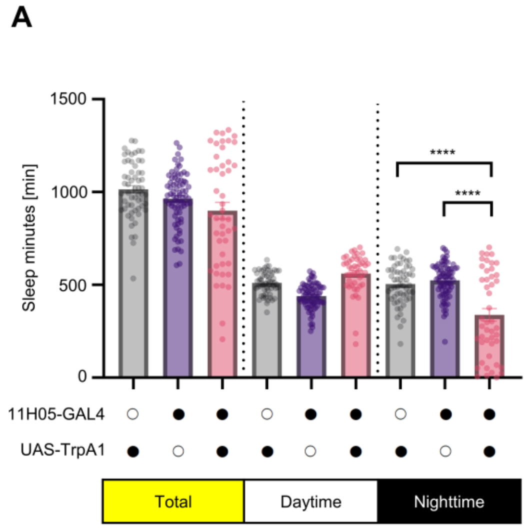

Figure S3. Validation of sleep deprivation in male flies.
(A, B) Sleep durations (daytime + nighttime combined, daytime only, nighttime only) of male flies with temperature-dependent activation of (A) GMR11H05+ neurons (n = 46-76) and (B) GMR60D04+ neurons (n = 34-58), measured on the 7th day of DAM assay. Filled circles indicate the presence of the corresponding transgene (GAL4 or UAS); open circles indicate the absence. Data are presented as mean ± s.e.m. Welch's ANOVA with Dunnett's T3's post hoc test is used. ***$P < 0.001$; ****$P < 0.0001$.

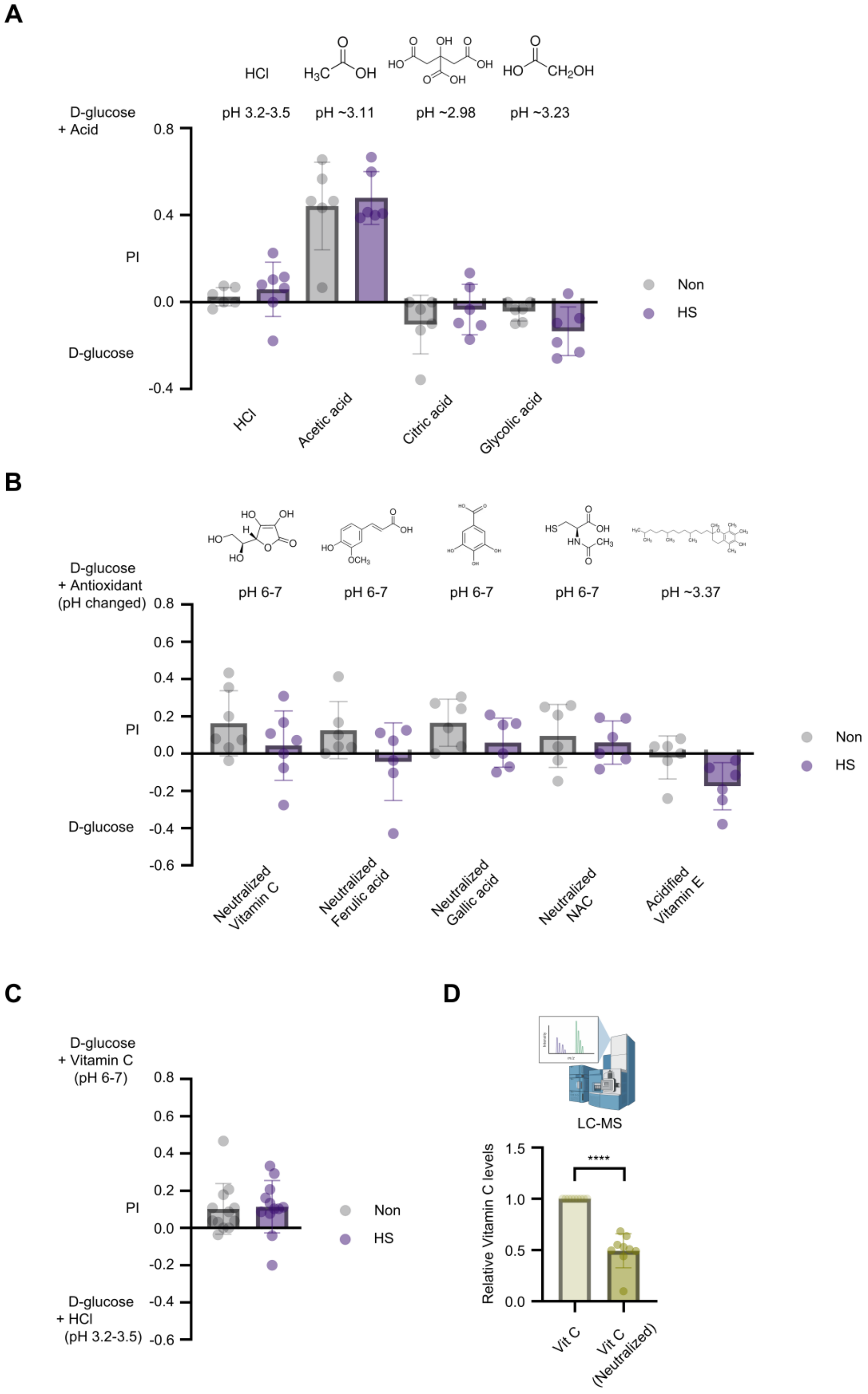
A
HCl
pH 3.2-3.5
pH ~3.11
pH ~2.98
pH ~3.23
D-glucose
+ Acid
PI
D-glucose
0.8
0.4
0.0
-0.4
HCl
Acetic acid
Citric acid
Glycolic acid
Non
HS
B
pH 6-7
pH 6-7
pH 6-7
pH 6-7
pH ~3.37
D-glucose
+ Antioxidant
(pH changed)
PI
D-glucose
0.8
0.6
0.4
0.2
0.0
-0.2
-0.4
-0.6
Neutralized Vitamin C
Neutralized Ferulic acid
Neutralized Gallic acid
Neutralized NAC
Acidified Vitamin E
Non
HS
C
D-glucose
+ Vitamin C
(pH 6-7)
PI
D-glucose
+ HCl
(pH 3.2-3.5)
0.8
0.6
0.4
0.2
0.0
-0.2
-0.4
-0.6
Non
HS
D
LC-MS
Relative Vitamin C levels
1.5
1.0
0.5
0.0
****
Vit C
Vit C (Neutralized)

Figure S4. Heat-stressed flies are not attracted to neutral vitamin C or acidic substances.

(A) Two-choice preferences (D-glucose versus D-glucose with HCl or 2 mM other acid) in heat-stressed flies (n = 6-7). (B) Two-choice preferences (D-glucose versus D-glucose with pH-adjusted antioxidant at 2 mM) of heat-stressed flies (n = 6-7). (C) Two-choice preferences (acidified D-glucose versus D-glucose with neutral 2 mM vitamin C) of heat-stressed flies (n = 12). (D) LC-MS measurement of vitamin C in neutralized pH (about pH 6-7) (n = 9). Data are presented as mean ± s.d. Unpaired two-tailed t-test is used. **** $P < 0.0001$.

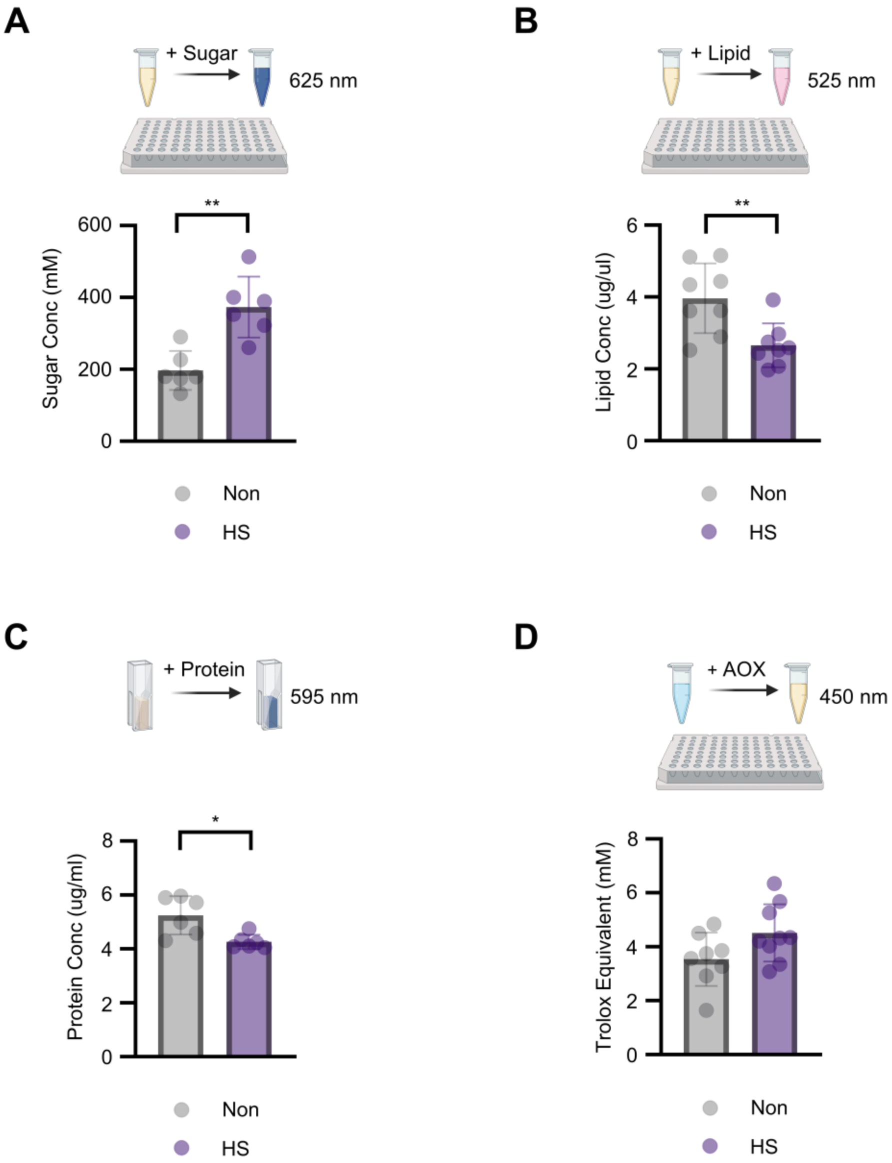

Figure S5. Heat shock results in the alteration of the hemolymph composition in flies.

(A-D) Measurements of macronutrient levels and antioxidant capacity in the hemolymph of heat-stressed flies. (A) sugar concentration (n = 6), (B) lipid concentration (n = 8), (C) protein concentration (n = 6), (D) antioxidant capacity (n = 8-9) in hemolymph. Data are presented as mean ± s.d. Unpaired two-tailed t-test is used. **P* < 0.05; ***P* < 0.01.

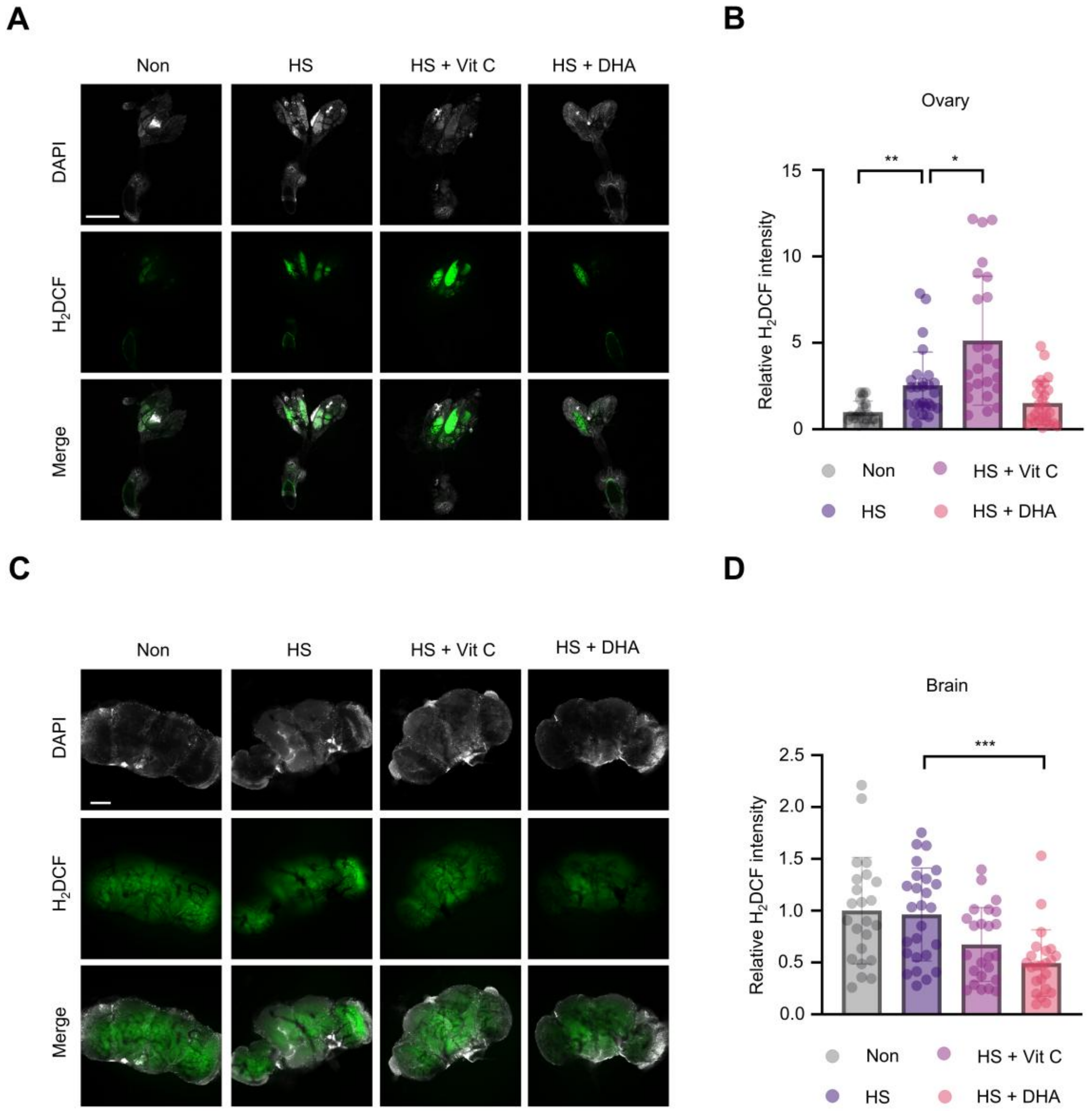

Figure S6. Measurement of ROS levels in the ovary and brain.

(A, C) Representative images of (A) the ovary and (B) the brain of heat-stressed flies that had been pre-fed 40 mM vitamin C, 40 mM DHA, or none for 4 days, stained with DAPI (gray) and H_2DCF (green). (B, D) Quantifications of H_2DCF intensities of (C) the ovary (n = 23-27) and (D) the brain (n = 23-26). Data are presented as mean ± s.d. Statistical analyses were performed as follows: Welch's ANOVA with Dunnett's T3's post hoc test for panel C; one-way ANOVA with Tukey's post hoc test for panel D. *$P < 0.05$; **$P < 0.01$; ***$P < 0.001$. Scale bar, 500 µm.

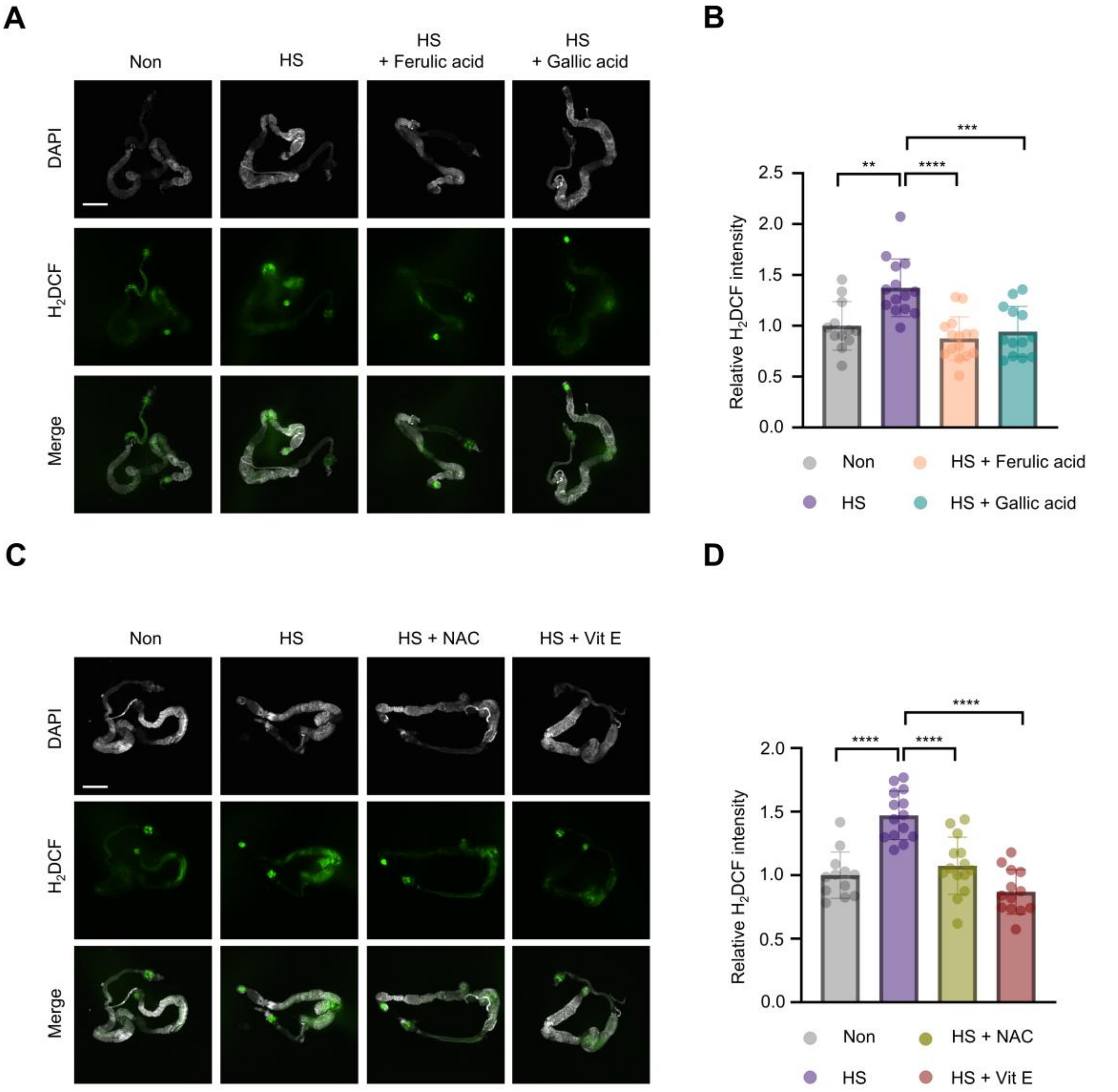

Figure S7. Measurement of ROS levels in the gut with pre-feeding of other antioxidants.

(A, C) Representative images of the gut of heat-stressed flies that had been pre-fed (A) 40 mM ferulic acid (FA), 40 mM gallic acid (GA), or none for 4 days and (B) 40 mM NAC, 40 mM vitamin E, or none for 4 days, stained with DAPI (gray) and H2DCF (green). (B, D) Quantifications of H_2DCF intensities corresponding to (A) (n = 12-15) and (C) (n = 12-14), respectively. Data are presented as mean ± s.d. One-way ANOVA with Tukey's post hoc test is used. ***P* < 0.01; ****P* < 0.001; *****P* < 0.0001. Scale bar, 500 μm.

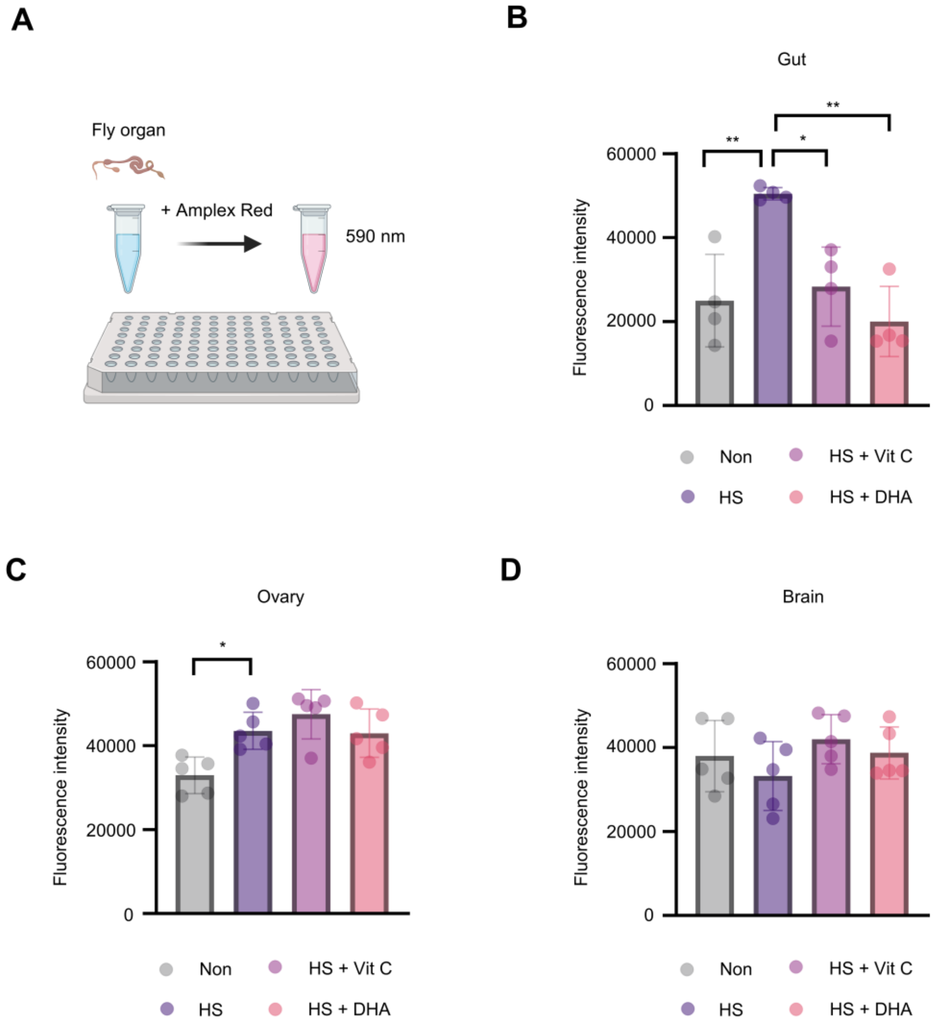

Figure S8. Alternative ROS measurement validates the original findings.
(A) Schematic illustration of the Amplex Red assay. (B-D) Fluorescence intensities stimulated by Amplex Red reaction in (B) the gut (n = 4), (C) ovary (n = 5), and (D) brain (n = 5) of heat-stressed flies that had been pre-fed 40 mM vitamin C, 40 mM DHA, and none for 4 days. Data are presented as mean ± s.d. One-way ANOVA with Tukey's post hoc test is used. *$P < 0.05$; **$P < 0.01$.
